# Exploring genetic variation that influences brain methylation in attention-deficit/hyperactivity disorder

**DOI:** 10.1038/s41398-019-0574-7

**Published:** 2019-10-03

**Authors:** Laura Pineda-Cirera, Anu Shivalikanjli, Judit Cabana-Domínguez, Ditte Demontis, Veera M. Rajagopal, Anders D. Børglum, Stephen V. Faraone, Bru Cormand, Noèlia Fernàndez-Castillo

**Affiliations:** 1 Departament de Genètica, Microbiologia i Estadística, Facultat de Biologia, Universitat de Barcelona, Barcelona, Catalonia Spain; 2 Centro de Investigación Biomédica en Red de Enfermedades Raras (CIBERER), Madrid, Spain; 3 Institut de Biomedicina de la Universitat de Barcelona (IBUB), Barcelona, Catalonia Spain; 4 Institut de Recerca Sant Joan de Déu (IR-SJD), Esplugues de Llobregat, Barcelona, Catalonia Spain; 5 The Lundbeck Foundation Initiative for Integrative Psychiatric Research, iPSYCH, Aarhus, Denmark; 6 Centre for Integrative Sequencing, iSEQ, Aarhus University, Aarhus, Denmark; 7 Department of Biomedicine - Human Genetics, Aarhus University, Aarhus, Denmark; 8 Departments of Psychiatry and Neuroscience and Physiology, SUNY Upstate Medical University, Syracuse, NY USA

**Keywords:** Comparative genomics, Epigenetics and behaviour, ADHD

## Abstract

Attention-deficit/hyperactivity disorder (ADHD) is a neurodevelopmental disorder caused by an interplay of genetic and environmental factors. Epigenetics is crucial to lasting changes in gene expression in the brain. Recent studies suggest a role for DNA methylation in ADHD. We explored the contribution to ADHD of allele-specific methylation (ASM), an epigenetic mechanism that involves SNPs correlating with differential levels of DNA methylation at CpG sites. We selected 3896 tagSNPs reported to influence methylation in human brain regions and performed a case-control association study using the summary statistics from the largest GWAS meta-analysis of ADHD, comprising 20,183 cases and 35,191 controls. We observed that genetic risk variants for ADHD are enriched in ASM SNPs and identified associations with eight tagSNPs that were significant at a 5% false discovery rate (FDR). These SNPs correlated with methylation of CpG sites lying in the promoter regions of six genes. Since methylation may affect gene expression, we inspected these ASM SNPs together with 52 ASM SNPs in high LD with them for eQTLs in brain tissues and observed that the expression of three of those genes was affected by them. ADHD risk alleles correlated with increased expression (and decreased methylation) of *ARTN* and *PIDD1* and with a decreased expression (and increased methylation) of *C2orf82*. Furthermore, these three genes were predicted to have altered expression in ADHD, and genetic variants in *C2orf82* correlated with brain volumes. In summary, we followed a systematic approach to identify risk variants for ADHD that correlated with differential *cis*-methylation, identifying three novel genes contributing to the disorder.

## Introduction

Attention-deficit/hyperactivity disorder (ADHD) is a common neurodevelopmental disorder with a worldwide prevalence of around 5%^1^. Its main symptoms include inattention and/or hyperactivity-impulsivity (DSM-V)^2^. ADHD is among the most heritable psychiatric disorders, with about 76% of its etiology accounted by genetic risk factors^3^ and with single-nucleotide polymorphisms (SNPs) explaining around 22% of the phenotypic variance^4^. Furthermore, there is molecular evidence of shared genetic risk factors across many psychiatric disorders^5^. In ADHD, a recent genome-wide association study (GWAS) meta-analysis of 12 sample groups unraveled some of the specific genetic underpinnings of this polygenic disorder for the first time^4^. One of the challenges of GWAS is to establish the causal relationship between the associated genetic variants, especially those located outside genes, and the disorder. In this regard, the use of epigenetic information can improve the interpretation of functionality of non-coding genetic variation^6^. In addition, some studies have hypothesized the importance of sub-threshold variants derived from GWAS^7,8^, particularly those located in enhancer regions, with a potential impact on gene regulation^9,10^.

DNA methylation is one of the most stable epigenetic mechanisms, involving mainly cytosines of CpG dinucleotides. This mechanism plays an important role in the regulation of neurogenesis, differentiation, and brain development^11^. Furthermore, epigenetic alterations have been hypothesized to contribute to neurodevelopmental disorders^12^, including ADHD^13^, autism spectrum disorders (ASD)^14,15^, or borderline personality disorder^16^.

DNA methylation can be complementary if it involves both alleles, or non-complementary when it affects only one allele, as in chromosome X inactivation in females or allele-specific methylation (ASM)^6^. ASM is a common mechanism by which single nucleotide variants determine differential methylation levels of CpG sites. ASM can alter promoter activity, leading to allele-specific expression^17^ in combination with other still quite unknown factors, such as environmental effects^6^. It is quantitative and heterogeneous across tissues and individuals^6^. The environment affects DNA methylation leading to changes in gene regulation, although the underlying mechanism is still not well understood^18^. It has been suggested that, during embryonic development, ASM regions could be especially sensitive to environmental effects^6^. Investigating SNPs that display ASM could help to identify risk variants for common diseases, including neuropsychiatric disorders^19^, as shown by recent studies of bipolar disorder (BD) and schizophrenia^10,20^.

The present study investigated the possible contribution of ASM to ADHD using data from the largest GWAS meta-analysis performed to date in ADHD^4^. We also assessed its possible effect on gene expression and on brain volumes to identify new genes contributing to the disorder.

## Materials and methods

### Selection of ASM SNPs

SNP selection was made based on the results of two previous studies^21,22^, which identified ASM variants in multiple brain regions of post-mortem human samples. Gibbs et al.^21^, considered four brain regions (cerebellum, frontal cortex, caudal pons, and temporal cortex) of 150 subjects and Zhang et al.^22^, used only the cerebellum of 153 subjects. Gibbs et al.^21^, unlike Zhang et al.^22^, excluded those sequences of probes with significant correlation with methylation that contained polymorphisms. To discard possible artifacts in our results, we checked and confirmed that none of the probes used to detect the six highlighted CpG sites target genomic regions with SNP variants. The genotyping platforms used in the two studies were different (Gibbs et al.^21^ used Infinium HumanHap550 Beadchips and Zhang et al.^22^ used Affymetrix GeneChip Mapping 5.0K Array). Both studies evaluated DNA methylation using the HumanMethylation27 Beadchips, and performed linear regression analyses by PLINK^23^ to determine the correlation between each SNP and methylation of any CpG site^21,22^. Zhang et al.^22^, unlike Gibbs et al.^21^ applied quantile normalization to the residuals prior to the linear regression analyses.

**Fig. 1:**
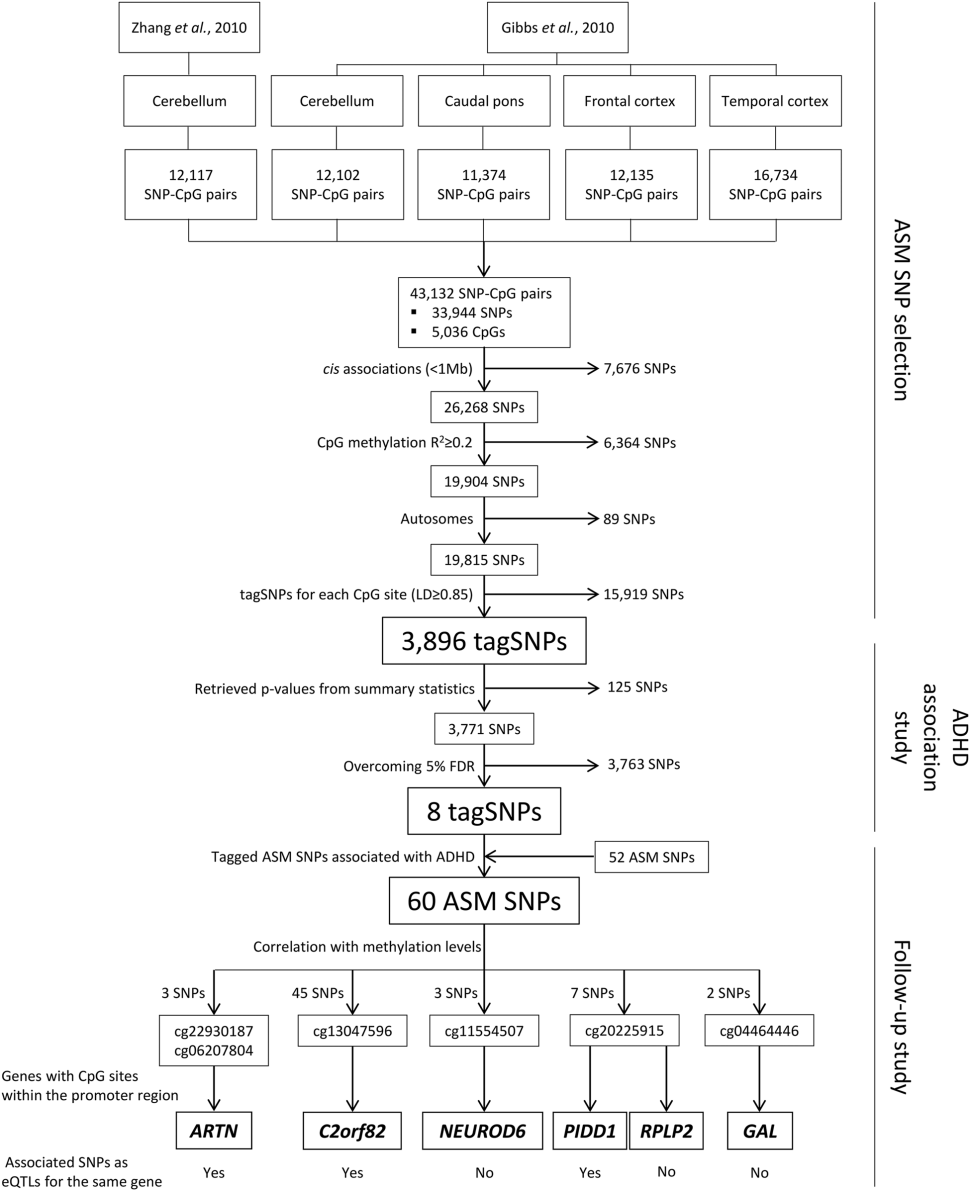
Selection of allele-specific methylation (ASM) SNPs and association results obtained for ASM variants in ADHD. SNPs tested in the ADHD GWAS meta-analysis and multiple testing correction. SNPs correlating with differential methylation of CpG sites and eQTLs in brain regions (only for genes in which the CpG site lies <5 kb from the transcription start site) are depicted

In the study by Zhang et al.^22^, a total of 12,117 SNP–CpG pairs associations were reported in cerebellum, and Gibbs et al.^21^ listed a total of 12,135 SNP–CpG pairs in frontal cortex, 11,374 in caudal pons, 16,734 in temporal cortex, and 12,102 in cerebellum (Fig. 1). We combined the information from both studies and obtained a total of 43,132 SNP–CpG pairs involving 33,944 different SNPs and 5306 CpG sites (Fig. 1). We considered all the ASM SNPs from all the tissues in the two studies, as there are multiple SNP–CpG pairs in common between them (Fig. S1).

We subsequently applied different filters to generate a sub-list of 3896 SNPs (Figs. 1 and S2) out of these 33,944 variants to minimize redundancy: associations in *cis* between the SNP and the CpG site, correlation of the SNP with methylation levels of the CpG (*R*^2^) ≥ 0.2, as performed in Gibbs et al. (2010)^21^. We considered only autosomal SNPs and selected tagSNPs for each CpG site (*r*^2^ ≥ 0.85), by assessing linkage disequilibrium (LD) with Haploview software^24^ using the Central European (CEU) reference panel from 1000 Genomes Project Phase 3^25^.

### Case-control GWAS datasets

We explored the selected ASM SNPs in the summary statistics from a meta-analysis of 11 independent GWAS of ADHD conducted by the Psychiatric Genomics Consortium (PGC) and iPSYCH. This case-control study investigated 8,047,420 markers in 20,183 cases and 35,191 controls from Europe, USA, Canada, and China, with patients diagnosed according to the criteria detailed in Demontis et al. (2019)^4^.

### Statistical analysis

To test whether risk variants for ADHD are enriched in ASM SNPs, we carried out an enrichment analysis using the Fisher’s exact test in R^26^ at *p*-value thresholds ranging from 5E−02 to 5E−08 considering the total number of ASM SNPs available from the ADHD GWAS meta-analysis^4^ (32,884 out of 33,944 SNPs).

From our selection of 3896 ASM tagSNPs, we could retrieve information on the association with ADHD of 3771 SNPs (96.8%) that were present in the summary statistics of the ADHD GWAS meta-analysis (Fig. 1)^4^. False discovery rate (FDR) was applied to correct for multiple testing. We used the *q*-value package for R^27^ and obtained a threshold *p*-value of 6.78E−05 corresponding to a 5% FDR. CpG sites highlighted by SNPs that were significant at this FDR threshold were followed-up in further analyses (Fig. 1). Additionally, we performed corrections for multiple testing, using Bonferroni and Genetic type 1 Error Calculator (GEC) methods (http://grass.cgs.hku.hk/gec/)^28^. The Bonferroni-corrected threshold was set at *p* ≤ 1.32E−05, which considered all the SNPs and tests to be independent (0.05/3771 SNPs). The GEC established the significance threshold at 1.98E−05, which addressed multiple testing for the set of 3771 dependent SNPs by estimating the independent number of tests. The LD between SNPs was calculated according to the 1000 Genomes EUR reference data^25^.

Finally, we considered and retrieved *p*-values of those tagged ASM SNPs in high LD (*r*^2^ ≥ 0.85) with the previous ones that also correlated in *cis* with the methylation levels of the same CpG sites (*R*^2^ ≥ 0.2) (Fig. 1).

### Functional annotation of associated ASM SNPs

We applied four methods to obtain information about the possible functional impact of the ASM SNPs that were associated with ADHD. First, we evaluated the presence of possible enhancer or promoter regions using the Haploreg v4.1 tool^29^. To do this, we considered histone modifications related to enhancer regions (H3K4me1 and H3K27ac) and promoters (H3K4me3 and H3K9ac) of 10 different brain regions (hippocampus middle, substantia nigra, anterior caudate, cingulate gyrus, inferior temporal lobe, angular gyrus, dorsolateral prefrontal cortex, germinal matrix, and male and female fetal brain). Second, we evaluated the effect on gene expression through an eQTL analysis using GTEx data (Release V7)^30^. We considered eQTL information for all available brain tissues: amygdala, anterior cingulate cortex (BA24), caudate basal ganglia, cerebellar hemisphere, cerebellum, cortex, frontal cortex (BA9), hippocampus, hypothalamus, nucleus accumbens basal ganglia, putamen basal ganglia, spinal cord cervical c-1, and substantia nigra. Third, we considered all the SNPs, not only ASMs, located within ±1 Mb from the transcription start site (TSS) of each gene to infer if the genetically determined expressions of genes of interest correlated with ADHD. This analysis was carried out using MetaXcan^31^, the input being the summary statistics of the ADHD GWAS meta-analysis^4^ and prediction models trained with RNA-Seq data of 10 GTEx^30^ brain tissues and CommonMind^32^ dorsolateral prefrontal cortex. The SNP covariance matrices were generated using the 1000 Genomes Project Phase 3^25^ EUR genotypes of the prediction model SNPs. Bonferroni correction for multiple testing was considered (*p* ≤ 2.27E−03; 0.05/22 tests). Finally, we examined the possible influence of the identified variants on subcortical brain structures. We obtained the summary statistics of a GWAS meta-analysis of eight MRI volumetric measures (nucleus accumbens, amygdala, caudate nucleus, hippocampus, pallidum, putamen, and thalamus) produced by the Enhancing Neuro Imaging Genetics through Meta-Analysis (ENIGMA) consortium^33^. This ENIGMA2 discovery sample included 13,171 subjects of European ancestry and contained association results between seven million markers and variance in the volumes of the mentioned structures^33^; we applied the Bonferroni correction (*p* ≤ 1E−03; 0.05/50 SNPs).

## Results

We investigated the possible association with ADHD of SNPs that show ASM in brain regions. Starting from two previous studies^21,22^ that describe ASM in brain tissues we obtained 43,132 SNP–CpG pairs involving 33,944 SNPs and 5306 CpG sites (Figs. 1 and S1). Genetic risk variants for ADHD are enriched in those ASM SNPs, as observed through enrichment analysis at different association *p*-value thresholds (Table S1).

**Fig. 2:**
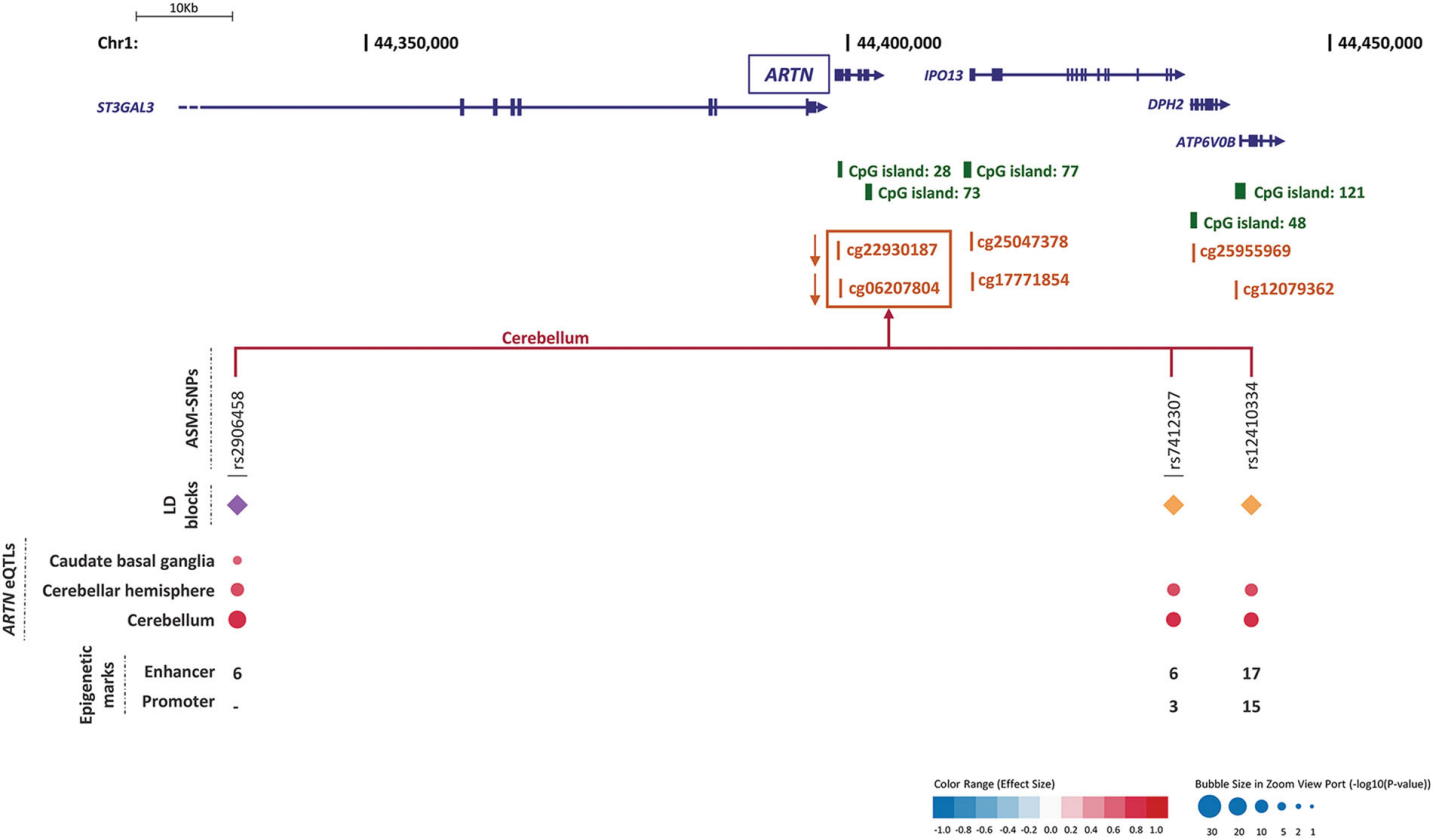
Genomic context of ASM variants, and methylation and eQTL information for cg22930187 and cg06207804. Genes are depicted in dark blue, showing the direction of transcription with an arrow; CpG sites inspected in the reference studies appear in brown; framed CpG sites indicate those sites showing differential levels of methylation for the associated ASM SNPs, and brown arrows indicate the effect on methylation of the ADHD risk variants, with indication of the brain regions where the ASMs were described. The tagSNPs are underscored. The colored rhombuses show the LD blocks present in each region. The colored dots for eQTLs indicate the effect on gene expression of the ADHD risk allele, according to the legend (red: over-expression, blue: under-expression). The number of enhancer (H3K4me1 and H3K27ac) and promoter (H3K4me3 and H3K9ac) histone marks found in the different brain areas are displayed for each SNP. ‘-' indicates no known enhancer or promoter histone marks

**Fig. 3:**
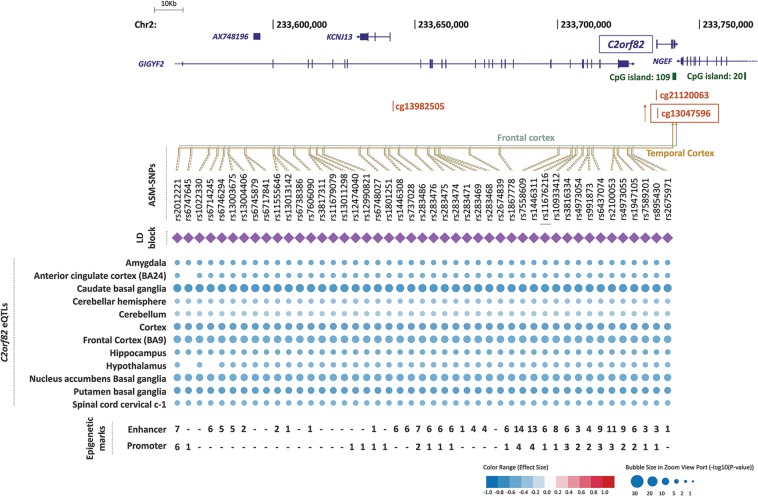
Genomic context of ASM variants, and methylation and eQTL information for cg13047596. Genes are depicted in dark blue, showing the direction of transcription with an arrow; CpG sites inspected in the reference studies appear in brown; framed CpG sites indicate those sites showing differential levels of methylation for the associated ASM SNPs, and brown arrows indicate the effect on methylation of the ADHD risk variants, with indication of the brain regions where the ASMs were described. The tagSNPs are underscored. The colored rhombuses show the LD blocks present in each region. The colored dots for eQTLs indicate the effect on gene expression of the ADHD risk allele, according to the legend (red: over-expression, blue: under-expression). The number of enhancer (H3K4me1 and H3K27ac) and promoter (H3K4me3 and H3K9ac) histone marks found in the different brain areas are displayed for each SNP. ‘-' indicates no known enhancer or promoter histone marks

**Fig. 4:**
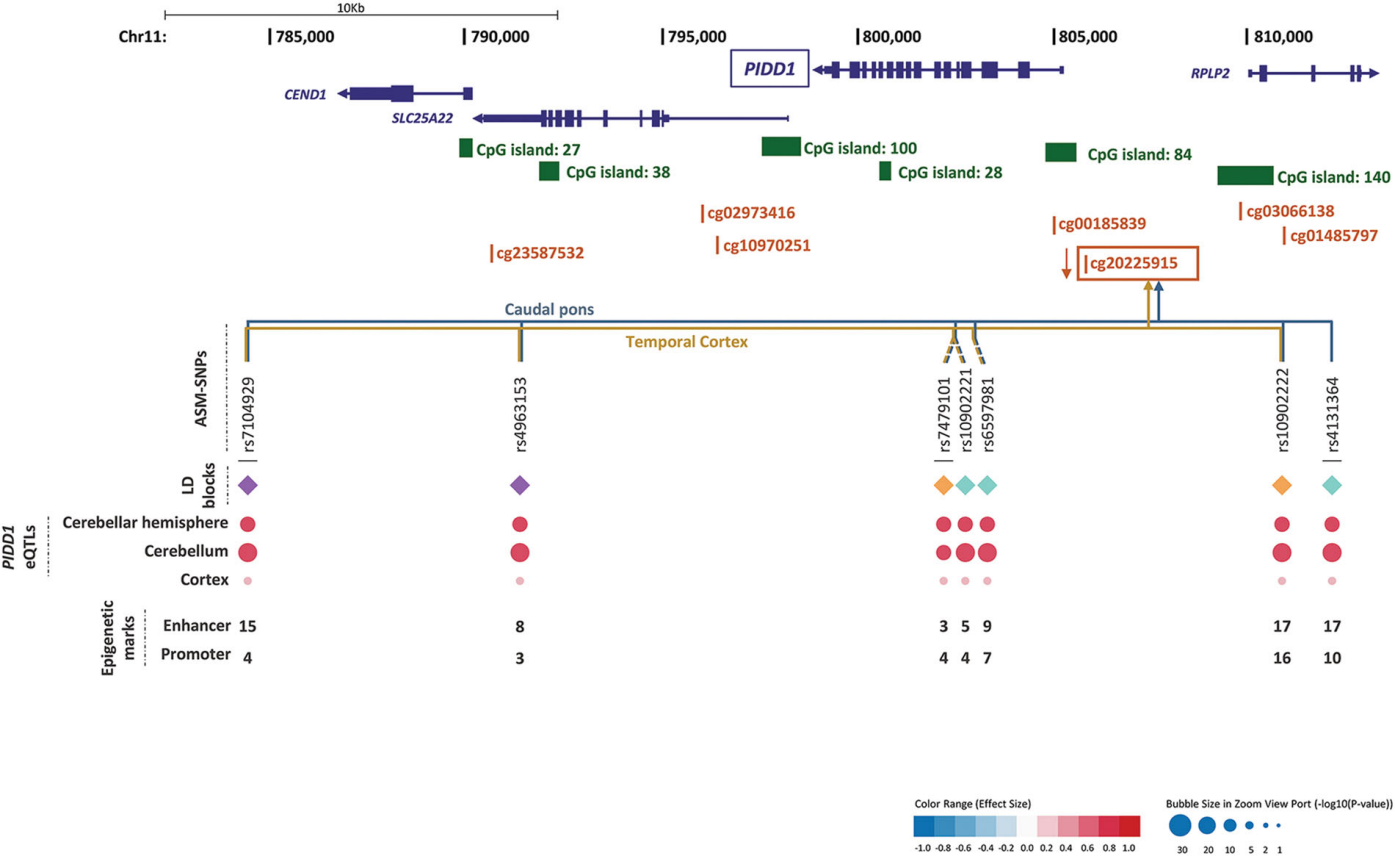
Genomic context of ASM variants, and methylation and eQTL information for cg20225915. Genes are depicted in dark blue, showing the direction of transcription with an arrow; CpG sites inspected in the reference studies appear in brown; framed CpG sites indicate those sites showing differential levels of methylation for the associated ASM SNPs, and brown arrows indicate the effect on methylation of the ADHD risk variants, with indication of the brain regions where the ASMs were described. The tagSNPs are underscored. The colored rhombuses show the LD blocks present in each region. The colored dots for eQTLs indicate the effect on gene expression of the ADHD risk allele, according to the legend (red: over-expression, blue: under-expression). The number of enhancer (H3K4me1 and H3K27ac) and promoter (H3K4me3 and H3K9ac) histone marks found in the different brain areas are displayed for each SNP. ‘-' indicates no known enhancer or promoter histone marks

We detected some overlaps and redundancies between studies and tissues (Fig. S1), so we performed a selection process ending up with a list of 3896 ASM tagSNPs (Figs. 1 and S2). Eight ASM tagSNPs were significantly associated with ADHD after correcting for multiple comparisons (5% FDR, *p* ≤ 6.78E−05) (Fig. 1 and Table S2). These eight SNPs correlated with differential methylation at six CpG sites in *cis* (three for cg20225915, two for both cg22930187 and cg06207804, and one for each of cg13047596, cg11554507, and cg04464446) in different brain areas (Figs. 2–4 and regional associational plots Figs. S3–S10, Table S2). Three of the eight ASM tagSNPs remained associated with ADHD after applying the Bonferroni and GEC corrections, all correlating with differential methylation at the cg20225915 site (Table S2).

As considering only tagSNPs may overlook true causal variants, we retrieved association results from all the 52 ASM SNPs tagged by the previous ones (LD; *r*^2^ ≥ 0.85), ending up with 60 variants in eight LD blocks that show association with ADHD and correlate with methylation levels at six CpG sites (Figs. 2–4 and S11–S15 and Table S3). We also selected, for each LD block, the SNP showing the highest number of functional annotations (Table 1), as a putative causal SNP.

**Table 1:** Selection of putative causal ASM SNPs associated with ADHD according to functional annotations

SNP	Association with ADHD^a^		Effect on methylation^b^	Epigenetic marks^c^		Effect on expression (GTEx data)^d^	Effect on brain volumes^e^
	Risk allele	*p*-value		Enhancer	Promoter		
rs2906458	G	3.01E−05	↓ cg22930187, ↓ cg06207804	6	0	***↑ ARTN***	–
rs12410334	A	2.87E−05		17	15		–
rs7558609	A	7.06E−05	↑ cg13047596	14	4	***↓ C2orf82***	↑ NAc ↑ CN
rs4140961	A	6.05E−05	↓ cg11554507	3	0	–	↑ T
rs7104929	G	7.89E−06	↓ cg20225915	15	4	***↑ PIDD1*** ↓ *PNPLA2*	?
rs10902222	T	2.03E−06		17	16		–
rs4131364	A	1.60E−06		17	10		–
rs1054252	G	3.86E−05	↑ cg04464446	4	0	↑ *MRPL21,* ↑ *MRGPRD* ↓ *IGHMBP2*	↓ NAc ↓ CN

*ASM*: Allele-specific methylation, *SNP*: single nucleotide polymorphism, *NAc*: nucleus accumbens, *CN*: caudate nucleus, *T*: thalamus. Risk allele: all alleles are reported in the forward strand; Underlined: significant associations between ASM tagSNPs and ADHD overcoming Bonferroni correction for multiple testing and *p*-value threshold determined using independent number of tests (GEC); ↑: Hypermethylation/overexpression/increased brain volume; ↓: Hypomethylation/underexpression/decreased brain volume; “−”: No significant data for the SNP; “?”: No information available for the SNP; Enhancer: Number of H3K4me1 and H3K27ac marks; Promoter: Number of H3K4me3 and H3K9ac marks; In bold: genes with the reported CpG sites lying in their possible promoter region

^a^Data obtained from the PGC+iPSYCH ADHD GWAS meta-analysis^4^

^b^Described in Zhang et al. ^22^ and Gibbs et al. ^21^

^c^Histone marks found in brain areas

^d^eQTL information for brain tissues

^e^Data from the Enhancing Neuro Imaging Genetics through Meta-Analysis (ENIGMA) consortium^33^

Consistently, the direction of the effect of the risk alleles on methylation levels is the same for all the SNPs correlating with the same CpG site. Thus, the risk alleles correlate with decreased methylation of cg22930187, cg06207804, cg11554507 and cg20225915 and with increased methylation of cg13047596 and cg04464446^21,22^ (Figs. 2–4 and Tables 1, S2, and S3).

All six CpG sites are located in possible promoter regions (<5000 bp upstream from a TSS) of six genes (Table 1), all of them expressed in brain: *ARTN* (cg22930187 and cg06207804), *C2orf82* (cg13047596), *NEUROD6* (cg11554507), *PIDD1* (cg20225915), *RPLP2* (cg20225915), and *GAL* (cg04464446) (Figs. 2–4). Furthermore, 85% of the 60 ASM SNPs are located within a region with enhancer or promoter histone marks in at least one brain area (Figs. 2–4 and Tables S4–S8). All putative causal SNPs selected from each LD block lie within a region with histone marks, ranging from 3 to 17 in enhancer regions and from 4 to 16 in promoter regions (Table 1).

We subsequently assessed the possible effect of those 60 SNPs on gene expression and observed that 57 of them are eQTLs for different genes in brain regions (Table S3). Seven out of the eight putative causal SNPs are eQTLs in brain for at least one gene (Table 1). We focused on methylation in promoter regions, which is well established to inversely correlate with gene expression. The eQTLs for *ARTN*, *C2orf82*, and *PIDD1* correlated with methylation of CpG sites lying on their possible promoter regions, showing opposite directions for methylation and gene expression levels (Figs. 2–4 and Tables 1 and S3). The ADHD risk alleles are associated with increased expression of *ARTN* (in cerebellum and a subcortical region) and *PIDD1* (in cerebellum and cortex) and with decreased expression of *C2orf82* (in cortical, subcortical, and cerebellar regions) (Figs. 2–4 and Tables 1 and S3).

Consistently, the predicted direction of the effect on gene expression for these three genes is the same when we consider all variants within ±1 MB from the TSS (and not only the ASM SNPs). We found significant associations of gene expression with ADHD for the same three genes in multiple brain tissues using MetaXcan: *ARTN*, *PIDD1* showed increased expression (3.57 < *Z*-score <4.19 and 3.57 < *Z*-score < 5.37, respectively) and *C2orf82* with a decreased expression (−3.64 < *Z*-score < −3.07) (Table S7), all of them surviving the Bonferroni correction.

We also evaluated the correlation of the 60 ADHD-associated SNPs with subcortical brain volume changes in ENIGMA2 data. SNPs correlating with methylation at cg13047596 and at cg04464446 correlate with nucleus accumbens and/or caudate nucleus volumes, while the only SNP correlating with cg11554507, which is present in ENIGMA2, correlates with thalamus volume (Table S10). Three of the putative causal SNPs showed correlation with brain volumes (Table 1).

Interestingly, the majority of ASM SNPs that correlate with methylation levels of cg13047596, located in the promoter region of *C2orf82*, are eQTLs in brain for this gene, lie in a region with histone marks and correlate with volume changes of nucleus accumbens and caudate nucleus (Figs. 2–4, Tables 1 and S3–S8 and S10). All this functional evidence highlights the *C2orf82* gene as a good candidate for contributing to ADHD.

## Discussion

This study is the first comprehensive assessment of the contribution to ADHD of genetic variants altering methylation in the brain. We identified a total of 60 variants from eight LD blocks associated with ADHD that correlate with differential levels of methylation at six different CpG sites^21,22^ (Tables 1 and S3). All the variants from six out of the eight LD blocks alter the methylation of CpG sites lying at potential promoter regions and are also eQTLs for one of the following three genes in multiple brain regions: *ARTN*, *C2orf82*, and *PIDD1* (Figs. 2–4 and Tables 1 and S3). It is well known that DNA methylation in promoter regions inversely correlates with levels of gene expression^18^, and all these ASM variants associated with ADHD in our study are concordant with this statement.

The *ARTN* gene, highlighted by two tagSNPs, encodes Artemin, a ligand of the *GDNF* family (glial cell line-derived neurotrophic factor). Artemin supports the survival of sensory and sympathetic peripheral neurons in culture by interacting with GFRα3-RET and possibly also of dopaminergic neurons of the ventral mid-brain through activation of GFRα1-RET complex^34^. Gene Ontology (GO) pathways link it to key neurodevelopmental functions: axon guidance (GO:0007411), neuroblast proliferation (GO:0007405), and peripheral nervous system development (GO:0007422). Risk alleles for ADHD lead to an overexpression of *ARTN*. Previously, overexpression of *ARTN* has been studied in transgenic mice and been linked to an increase of neuron excitability that leads to hypersensitivity^35,36^. Another study in *ARTN* knockout mice reported aberrations in the sympathetic nervous system related to migration and axonal projection^37^. The *C2orf82* gene (also known as *SNORC*) was highlighted by one tagSNP and it encodes a proteoglycan transmembrane protein that is expressed in brain more than in other tissues^30^. Little is known about its function. Finally, *PIDD1* was highlighted by three tagSNPs. It is a cell life regulator gene and it has been linked to apoptotic and anti-apoptotic pathways. The PIDD protein initiates apoptosis as a component of the PIDDosome together with RAIDD (RIP-associated ICH-1/ECD3-homologous protein with a death domain) and procaspase-2^38^ and it also activates an anti-apoptotic pathway involving the transcription factor NF-κB in response to genotoxic stress^39^.

Alterations in the expression of these three genes (upregulation of *ARTN* and *PIDD1* and downregulation of *C2orf82*) in different brain regions seem to be related to ADHD. Interestingly, most of these regions are relevant for this disorder. Neuroimaging studies have implicated the cerebellum, subcortical and prefrontal regions in ADHD, suggesting a link to problems in the processing of temporal information^40^. Structural anomalies in the cerebellum have been reported in ADHD individuals through neuroimaging studies^41–43^. Cerebellar developmental trajectories and hippocampal volumes are linked to the severity of ADHD symptoms^44–46^. Structural and functional abnormalities in cerebellum and basal ganglia have been associated with motor impairments^47^, which are frequent in nearly half of ADHD cases^48^. Subcortical regions identified through our expression analyses have also been related to ADHD, for instance: (i) remarkably different shapes of caudate-putamen basal ganglia and smaller volumes have been reported in ADHD boys^49–52^; (ii) in adult males with ADHD, right caudate volume correlates with poor accuracy on sensory selection tasks^53^ and also with hyperactivity/impulsivity^54,55^; (iii) nucleus accumbens, caudate nucleus, putamen, amygdala, and hippocampus are structurally altered in the brains of ADHD patients^56^. Remarkably, all the ASM SNPs in the LD block for *C2orf82* with available information nominally correlate with increased volumes of nucleus accumbens and caudate nucleus subcortical regions. Also, the eQTL effect sizes of these SNPs are the largest for caudate basal ganglia, which volume correlates with the SNP genotype variation. There is evidence about the role in ADHD of cortical thickness, cortical volume and functional connectivity in the anterior cingulate cortex, a region involved in cognitive control, attention, affect and drive^57–63^. Furthermore, delayed cortical development, e.g. in prefrontal regions has been reported in ADHD patients^64,65^ and this appears to be stronger in ADHD children with below median intelligence quotient^66^. All the above mentioned fronto-subcortical structures and pathways are rich in catecholamines, the molecular targets in pharmacological treatments for ADHD^48,52,64,67^.

Interestingly, the methylation of cg20225915 has also been associated with *PIDD1* expression in peripheral blood^68^, turning it into a good candidate as a biomarker. The expression of *ARTN* was found to be altered in blood of major depressive disorder (MDD) patients^69^ and the *C2orf82* gene has been associated to schizophrenia^70,71^. Furthermore, *C2orf82* was highlighted in a cross-disorder GWAS of eight psychiatric conditions, including ADHD and schizophrenia^72^, with the rs778353 lead SNP, located 47 kb downstream from the gene, showing a genome-wide significant association with the phenotype. All three genes overlap with several CNVs that contribute to autism, intellectual disability or aggressive behavior, conditions often comorbid with ADHD (Table S11). It is noteworthy that some of the CNVs reported in *ARTN*, *C2orf82*, and *PIDD1* are related to brain-specific and overall developmental delay at both fetal and postnatal stages. Thus, it is reasonable to assume that altered expression of these genes might affect brain volumes and cognition. Overall, the fact that these genes have previously been related to neuropsychiatric disorders that are often comorbid with ADHD^73^ make them appealing candidates to be pursued.

*ARTN* is the only gene highlighted in our study that is present in one of the top regions reported in the ADHD GWAS meta-analysis^4^, although it did not contain SNPs surviving genome-wide significance. The GWAS findings in the region could be accounted for by one of several genes: *ST3GAL3*, *PTPRF*, *KDM4A*, *RP11-184I16.4*, *XR_246316.1*, *KDM4A-AS1*, and *SLC6A9*. *ST3GAL3* had the most signals. Although two of the reported ASM variants associated with ADHD are intronic to *ST3GAL3*, this gene was not highlighted in our study as none of the associated variants correlated with differential methylation of CpG sites near the *ST3GAL3* TSS (distance from the nearest CpG site: 197 kb) or were eQTLs for the gene in brain tissues. Instead, these SNPs correlated with a nearby gene, *ARTN*, both in terms of methylation and gene expression. This suggests the importance of finding functional connections between disease-associated SNPs and genes, besides considering the genes in the physical vicinity of variants. Furthermore, another of the highlighted genes, *PIDD1*, although not being among the top findings in the ADHD GWAS meta-analysis^4^, it is pointed out by the gene-based association analysis performed in the same study.

Genetic variants surpassing genome-wide significance in GWAS explain only a small part of the SNP-based heritability and associations not reaching the significance threshold also contribute to disease susceptibility^4,9^. An omnigenic model has been recently proposed suggesting that the sub-threshold variants could point at regulatory elements of core genes^7,8^. Indeed, a previous study on a cardiovascular cardiac phenotype reported that nominally significant associations are enriched in enhancer regions^9^, consistent with our findings. Therefore, although none of the variants that we identified in our study display genome-wide significant association with ADHD, they may contribute to the susceptibility to ADHD, as they do have a functional impact (methylation, expression, and in some cases brain structure) via genes that are expressed in brain.

Brain-specific ASM information has also been utilized to detect key genes and pathways in BD^20^. Also, a higher enrichment of brain ASM was observed in a schizophrenia GWAS in comparison to non-psychiatric GWAS^10^. This, together with the enrichment of ASM in ADHD-associated variants found in the present study, reinforces the rationale of utilizing ASM SNPs to highlight genes that are relevant to psychiatric disorders from GWAS data.

There are some strengths and limitations in our study that should be discussed. Strengths: (i) We used the largest GWAS meta-analysis of ADHD performed so far, including around 20,000 cases and 35,000 controls. (ii) The genetic variants identified as associated with ADHD have a functional impact on epigenetic regulation, expression or brain volumes. (iii) Two of the highlighted genes in this study, *ARTN* and *C2orf82*, have previously been associated with other psychiatric disorders. (iv) For two of the genes there is more than one LD block showing the same effect on CpG site methylation. (v) Our results are concordant with eQTL information that had been assessed in an independent sample, with all the SNPs showing the opposite effect on methylation of the promoter region and on the expression of a given gene in brain (more promoter methylation and less gene expression or vice versa), even for the different LD blocks from each region. Limitations: (i) We did not perform a follow-up study to replicate the association findings in an independent sample. (ii) The previous studies that we used for the selection of ASM SNPs were performed on different genotyping platforms that do not include all the existing SNPs in the genome, and therefore we could not test all possible ASMs. (iii) We only considered *cis*-associated ASM variants, which are the vast majority, although non-*cis* ASM also occurs. (iv) There is an overrepresentation of ASM SNPs from cerebellum compared to the other studied tissues.

To conclude, the present study points to the *ARTN*, *C2orf82*, and *PIDD1* genes as potential contributors to ADHD susceptibility. The identified risk variants have an impact on the methylation levels of different CpG sites located in promoter regions and they inversely correlate with expression of the corresponding genes in brain. This finding is supported by a prediction of increased expression of *ARTN* and *PIDD1*, and a decreased expression of *C2orf82* in ADHD. Moreover, variants correlating with methylation at cg13047596 (near *C2orf82*) influence the volumes of nucleus accumbens and/or caudate nucleus. Further studies are required to elucidate the mechanisms by which these genes contribute to ADHD.

## Supplementary information

Supplemental Material
