# Supplementary material for: Exploring genetic variation that influences brain methylation in attention-deficit/hyperactivity disorder
Source: Transl Psychiatry. 2019 Oct 3;9:242. doi: 10.1038/s41398-019-0574-7 (PMC6776507; doi:10.1038/s41398-019-0574-7)
Supplement: Supplementary file 1 — Supplemental Material [file 41398_2019_574_MOESM1_ESM.pdf]

Supplementary Material

EXPLORING GENETIC VARIATION THAT INFLUENCES BRAIN METHYLATION IN ATTENTION-DEFICIT/HYPERACTIVITY DISORDER

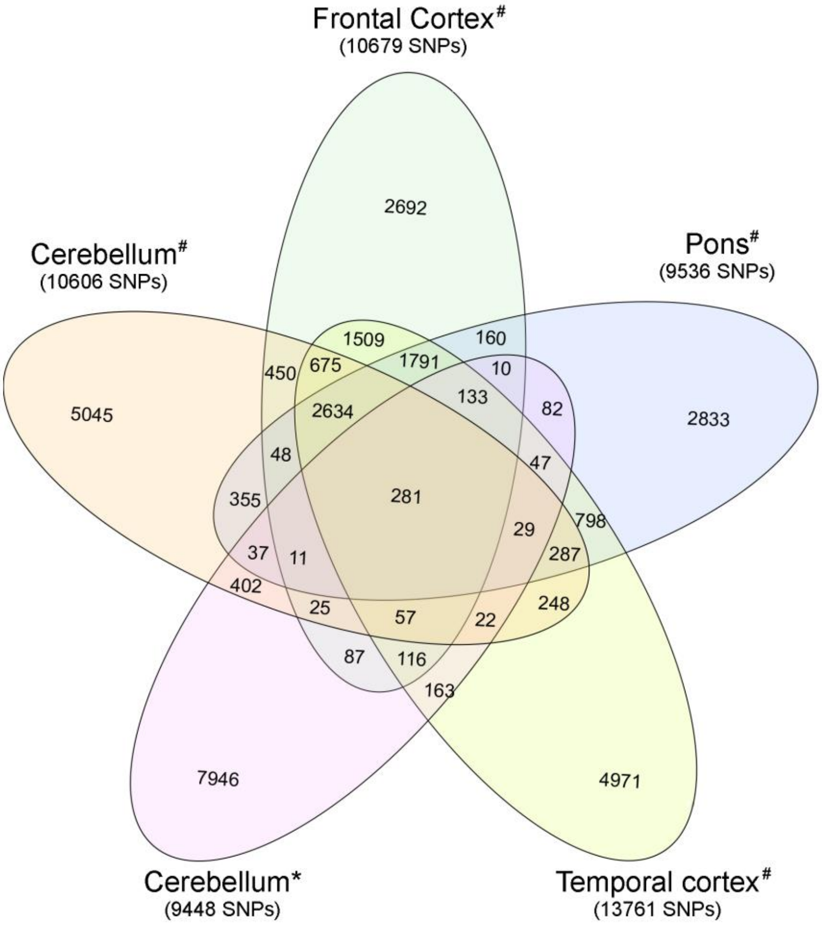

Figure S1. Venn diagram showing the overlaps among the initial 33,944 ASM SNPs selected in the different brain areas. ***Cerebellum from the study by Zhang *et al.* 2010. #Brain areas from the study by Gibbs *et al.* 2010. The overlap accounts for 31% of the ASM SNPs; 12.5% ASM SNPs are shared between two tissues, 9% are shared between three tissues, 8.5% are shared between four tissues and only 1% are shared between all the tissues. Sixteen percent of the 9,448 ASM SNPs identified by Zhang et al., 2010 overlap with the ASM SNPs identified in the Gibbs et al., 2010 study.

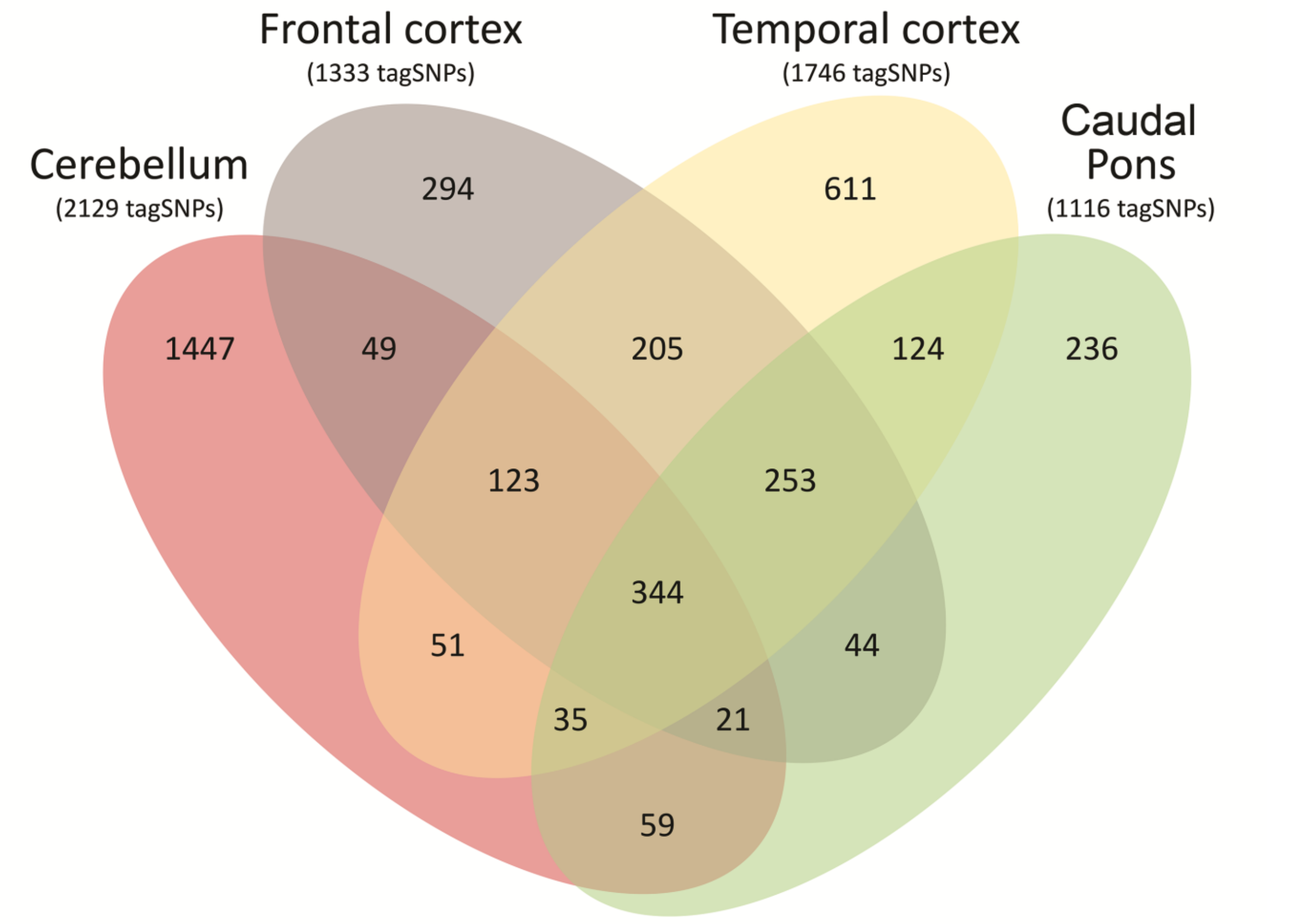

Figure S2. Venn diagram showing the overlaps among the 3,896 ASM tagSNPs selected in the different brain areas. The overlap accounts for 33.5% of tagSNPs; 13.5% tagSNPs are shared between two tissues, 11% are shared between three tissues and 9% are shared between all the tissues.

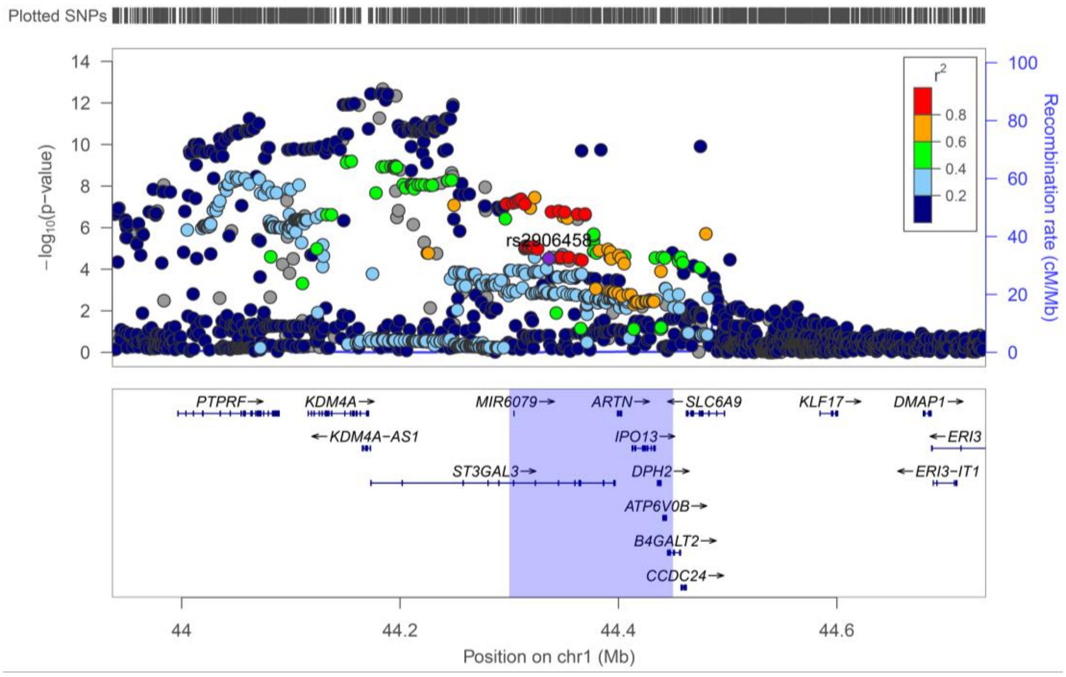

Figure S3. Regional association plot for rs2906458. The SNP represented in the regional plot is depicted in purple. Highlighted in blue: Region represented in Figure 2.

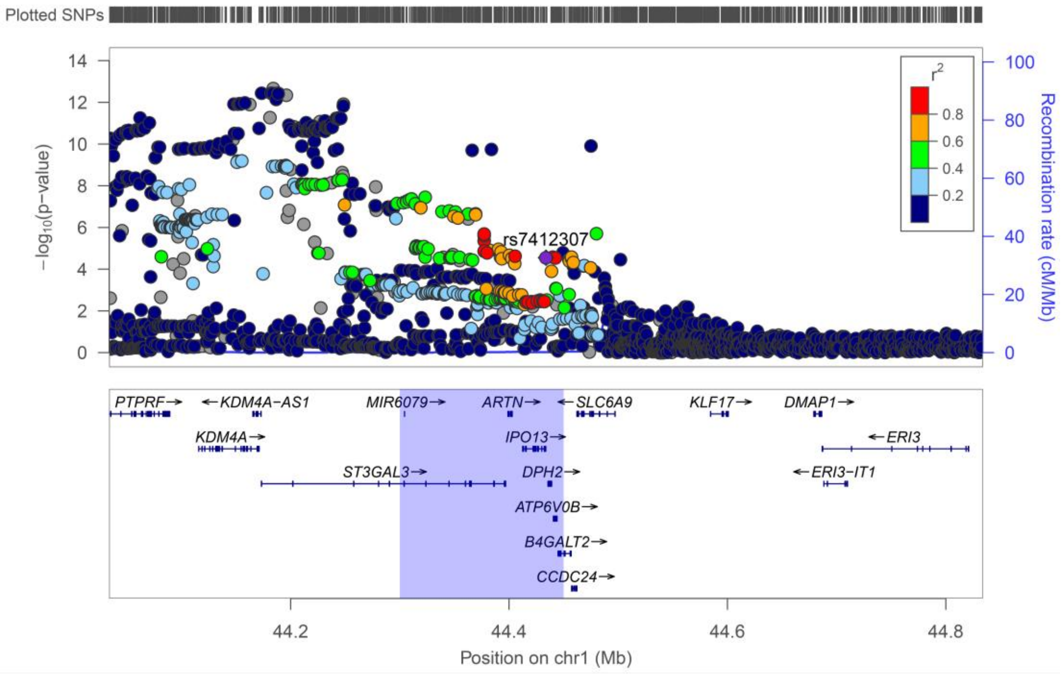

Figure S4. Regional association plot for rs7412307. The SNP represented in the regional plot is depicted in purple. Highlighted in blue: Region represented in Figure 2.

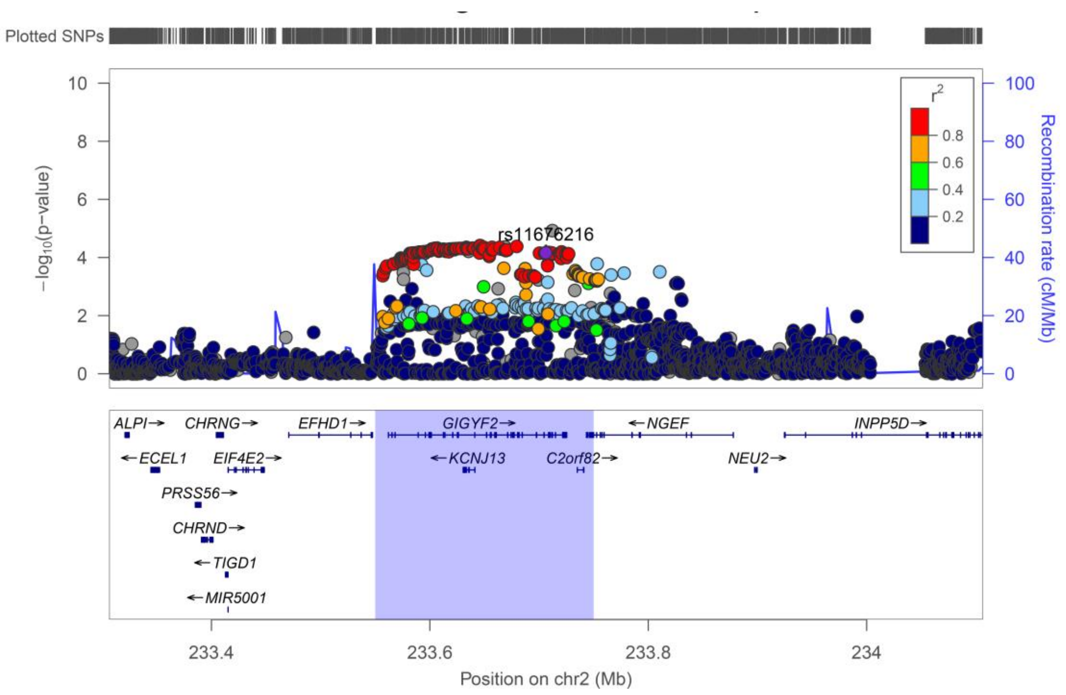

Figure S5. Regional association plot for rs11676216. The SNP represented in the regional plot is depicted in purple. Highlighted in blue: Region represented in Figure 3.

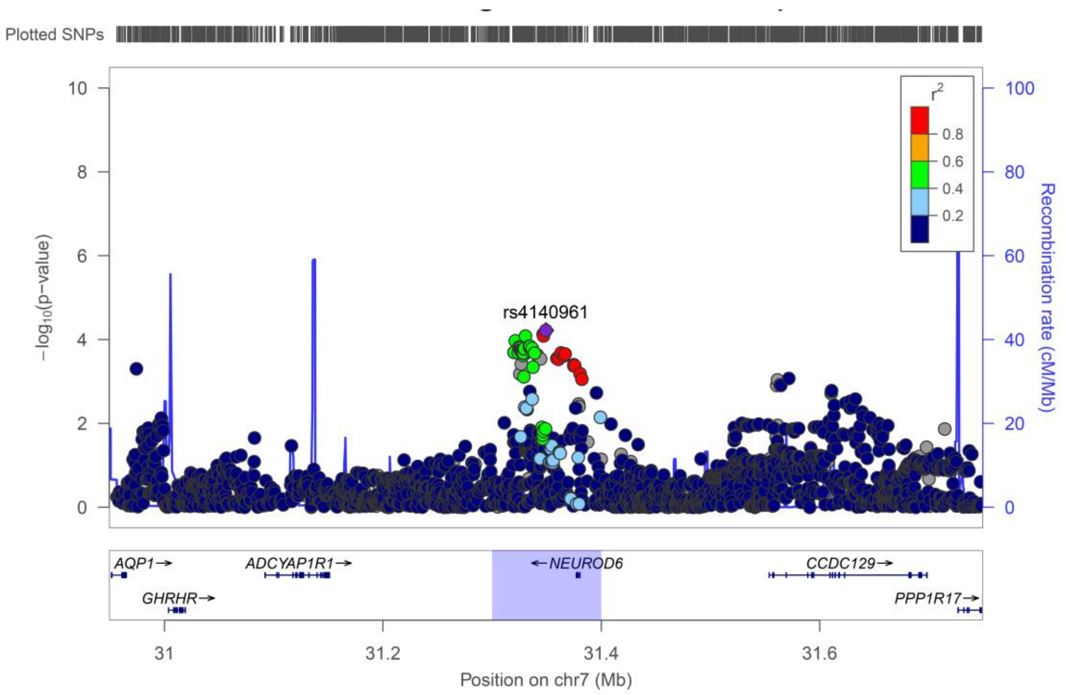

Figure S6. Regional association plot for rs4140961. The SNP represented in the regional plot is depicted in purple. Highlighted in blue: region containing the CpG site and the ASM-SNPs of interest.

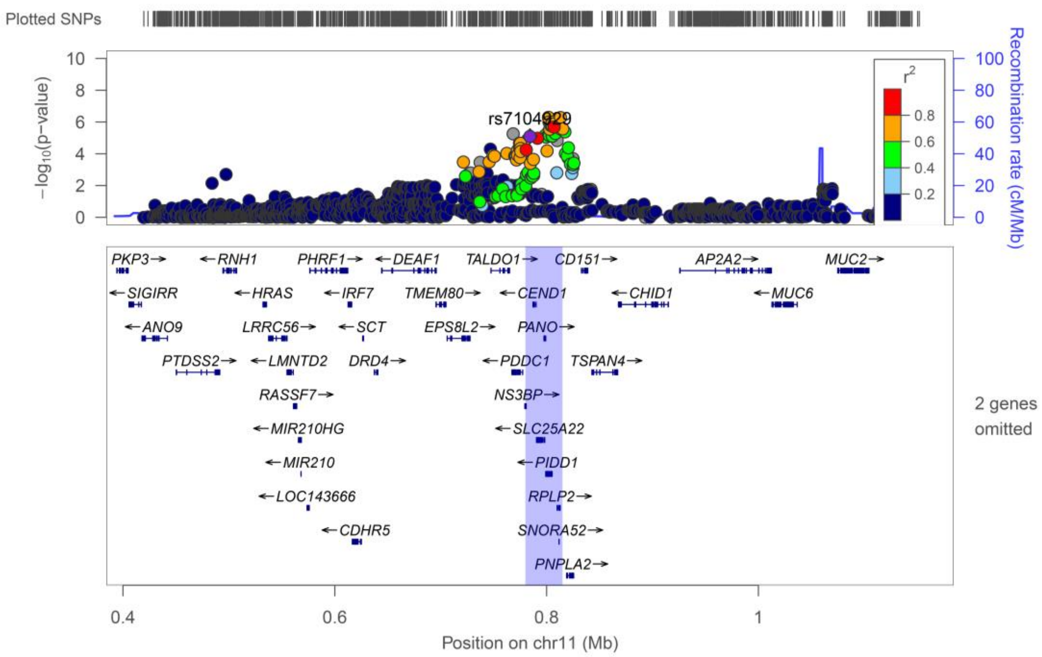

Figure S7. Regional association plot for rs7104929. The SNP represented in the regional plot is depicted in purple. Highlighted in blue: Region represented in Figure 4.

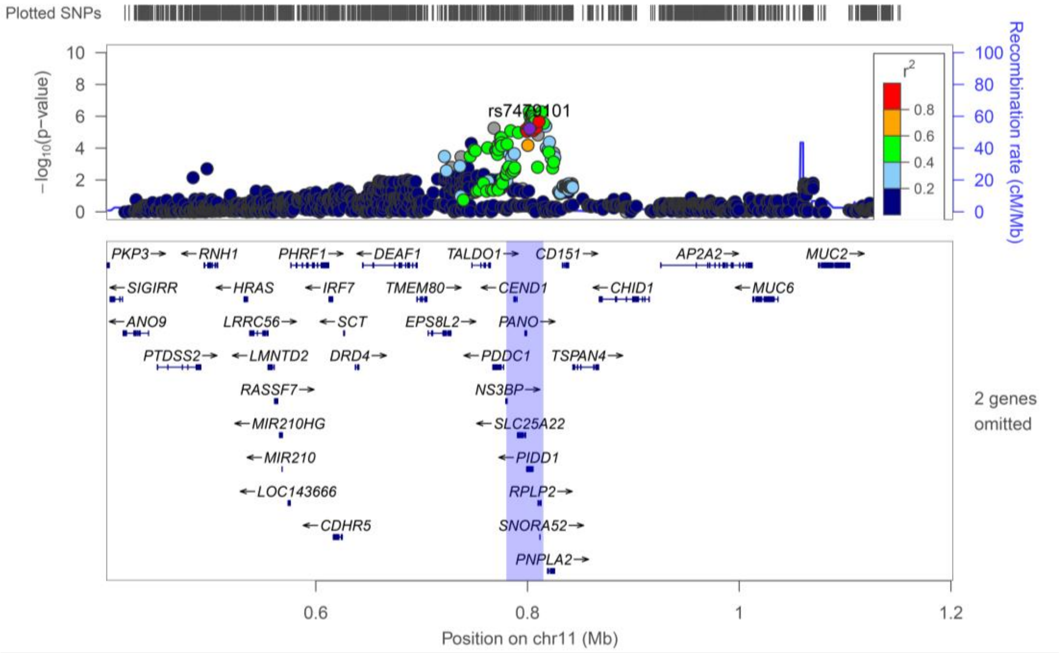

Figure S8. Regional association plot for rs7479101. The SNP represented in the regional plot is depicted in purple. Highlighted in blue: Region represented in Figure 4.

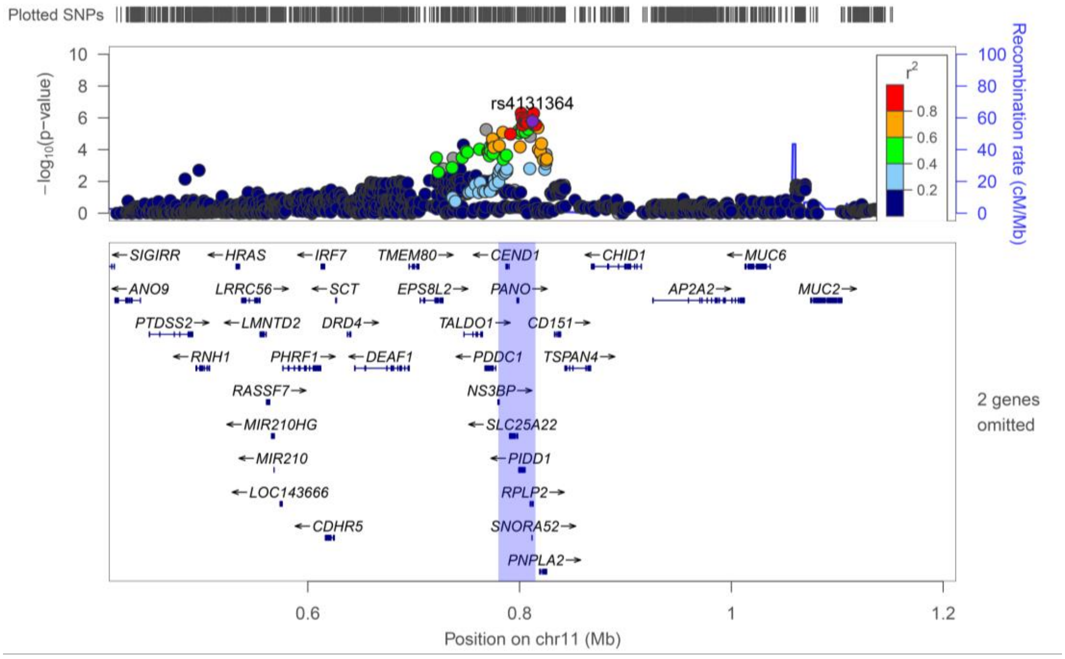

Figure S9. Regional association plot for rs4131364. The SNP represented in the regional plot is depicted in purple. Highlighted in blue: Region represented in Figure 4.

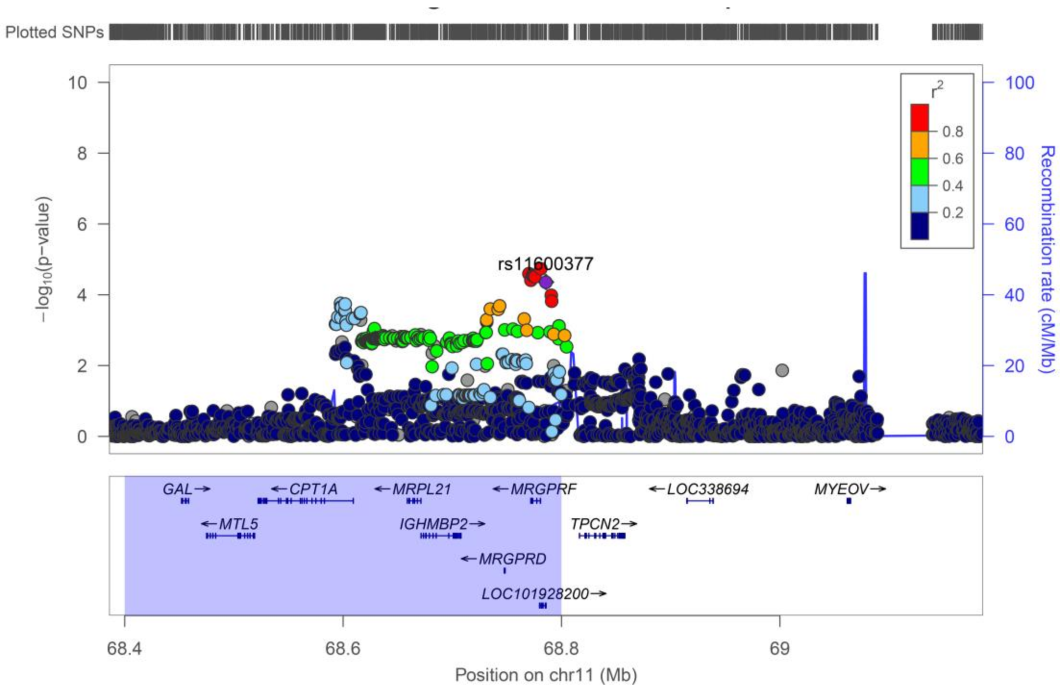

Figure S10. Regional association plot for rs11600377. The SNP represented in the regional plot is depicted in purple. Highlighted in blue: region containing the CpG site and the ASM-SNPs of interest.

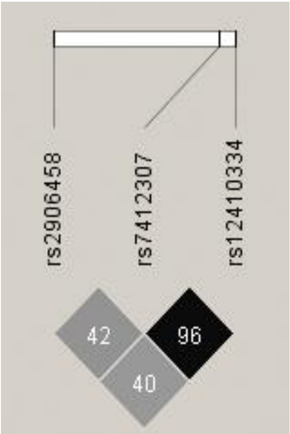

Figure S11. Linkage disequilibrium (LD, r^2) patterns between the three SNPs associated with ADHD that also correlate with differential methylation at two CpG sites, cg22930187 and cg06207804, located in the possible promoter region of *ARTN*.

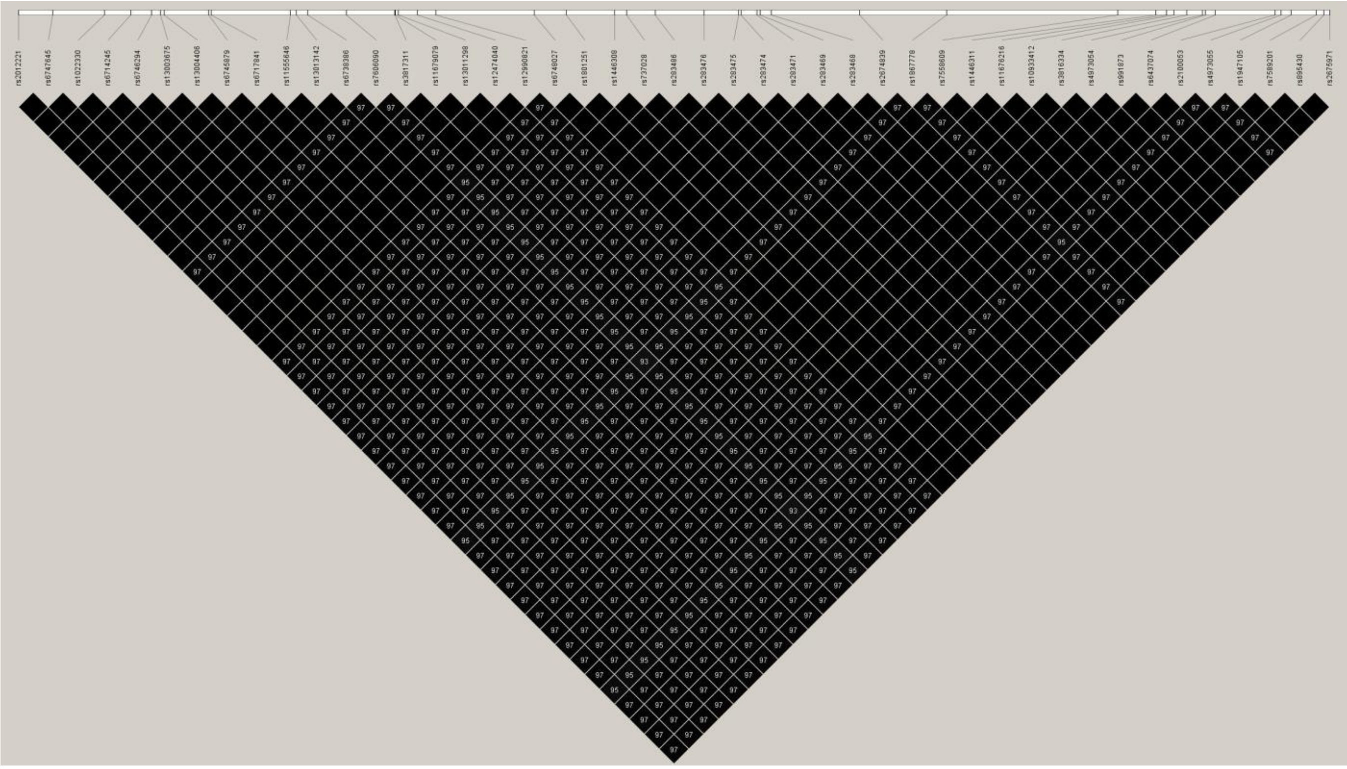

Figure S12. Linkage disequilibrium (LD, r^2) patterns between the 45 SNPs associated with ADHD that also correlate with differential methylation at the CpG site cg13047596, located in the possible promoter region of *C2orf82*.

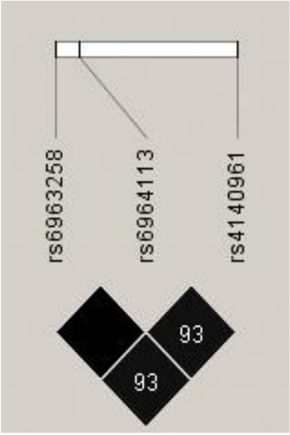

Figure S13. Linkage disequilibrium (LD, r^2) patterns between the three SNPs associated with ADHD that also correlate with differential methylation at the CpG site cg11554507, located in the possible promoter region of *NEUROD6*.

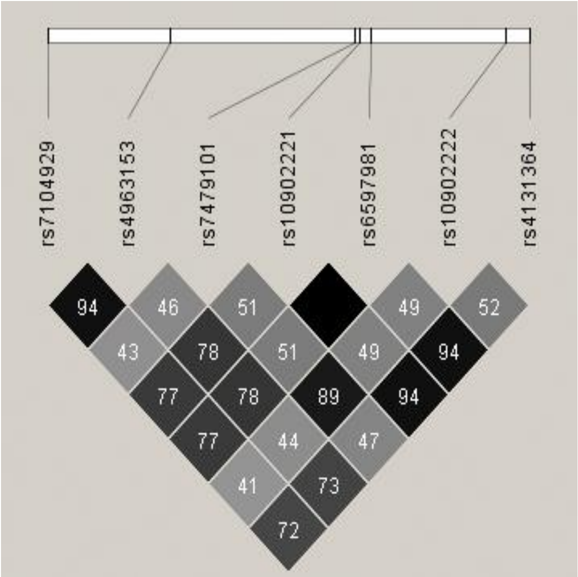

Figure S14. Linkage disequilibrium (LD, r^2) patterns between the 7 SNPs associated with ADHD that also correlate with differential methylation at the CpG site cg20225915, located in the possible promoter region of *PIDD1*.

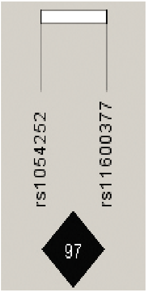

Figure S15. Linkage disequilibrium (LD, r^2) patterns between the two SNPs associated with ADHD that also correlate with differential methylation at the CpG site cg04464446, located in the possible promoter region of *GAL*.

Table S1. Enrichment analysis of ASM SNPs at different significance thresholds in the ADHD GWAS meta-analysis by Demontis et al. (2019).

**Significance Threshold**	**N SNPs**	**N ASM SNPs**	**p-value**	**OR**
5.00E-08	303	6	1.70E-03	4.92
5.00E-07	945	8	4.30E-02	2.08
5.00E-06	2,122	15	3.15E-02	1.74
5.00E-05	6,970	35	1.31E-01	1.23
5.00E-04	25,288	139	4.58E-04	1.35
5.00E-03	115,681	527	6.94E-03	1.12
5.00E-02	651,772	2790	5.54E-03	1.05

ASM: Allele-specific methylation; N SNPs: Significant SNPs in the ADHD GWAS meta-analysis for the corresponding significance threshold; N ASM SNPs: Significant ASM SNPs in the GWAS meta-analysis; Underlined: Significant enrichment of ASM SNPs in the list of ADHD-associated SNPs; OR: Odds ratio.

Table S2. ASM tagSNPs associated with ADHD.

	SNP information				[1]Association with ADHD				[2]Correlation with methylation			
			Alleles		Freq A1							
**SNP**	**Chr**	**Pos**	**A1**	**A2**	**Cases**	**Controls**	**OR**	**p-value**	**CpG site**	**Tissue**	**Effect**	**p-value**
rs2906458	1	44336389	A	G	0.74	0.756	0.94	3.01E-05	cg22930187	Crbl	↓	5.55E-10
									cg06207804	Crbl	↓	1.10E-12
rs7412307	1	44433864	C	G	0.185	0.172	1.07	2.82E-05	cg22930187	Crbl	↓	3.59E-11
									cg06207804	Crbl	↓	1.76E-15
rs11676216	2	233706368	T	C	0.646	0.654	0.95	6.78E-05	cg13047596	Tctx	↑	1.09E-10
										Fctx	↑	2.35E-10
rs4140961	7	31349352	A	G	0.597	0.592	1.06	6.05E-05	cg11554507	Pons	↓	8.45E-25
										Tctx	↓	4.72E-23
rs7104929	11	784340	C	G	0.512	0.526	0.94	**7.89E-06**	cg20225915	Pons	↓	8.17E-10
										Tctx	↓	1.51E-08
rs7479101	11	802115	A	G	0.317	0.33	0.93	**5.90E-06**	cg20225915	Pons	↓	2.15E-14
										Tctx	↓	3.37E-14
rs4131364	11	812188	A	G	0.517	0.502	1.07	**1.60E-06**	cg20225915	Pons	↓	2.49E-10
rs11600377	11	68785803	A	G	0.731	0.72	1.06	4.38E-05	cg04464446	Crbl	↑	3.14E-08

ASM: Allele-specific methylation; [1]Data obtained from the PGC+iPSYCH ADHD GWAS meta-analysis (Demontis et al., 2019); [2]Described in Zhang et al., 2010 and Gibbs et al., 2010; SNP: Single Nucleotide Polymorphism; Chr: Chromosome; Pos: Position (build hg19); A1: Allele 1; A2: Allele 2; All alleles are reported in the forward strand; Freq A1: Frequency of allele 1; OR: Odds Ratio (calculated on A1); Effect: Direction of the risk allele effect on DNA methylation levels; Underlined allele: Risk allele for ADHD; In bold: Significant associations for the association between ASM tagSNPs and ADHD p-values overcoming Bonferroni correction for multiple testing and p-value threshold determined using independent number of tests (GEC); Crbl: Cerebellum; Tctx: Temporal cortex; Fctx: Frontal cortex.

Table S3. ASM SNPs associated with ADHD.

SNP	TagSNP	LD (R^2)	Chr	Pos	Alleles A1	A2	[1] Association with ADHD Freq A1 cases	Freq A1 controls	OR	p-value	[2] ADHD risk allele effect on CpG methylation (ASM studies)	[3] ADHD risk allele effect on gene expression (GTEx data)
rs2906458*	rs2906458	1	1	44336389	A	<u>G</u>	0.74	0.756	0.94	3.01E-05		
rs7412307*	rs7412307	1	1	44433864	<u>C</u>	G	0.185	0.172	1.07	2.82E-05	↓ cg22930187, cg06207804	↑ *ARTN*
rs12410334		0.96	1	44442521	<u>A</u>	C	0.184	0.171	1.07	2.87E-05		
rs2012221	rs11676216	0.977	2	233566848	T	<u>C</u>	0.642	0.651	0.95	1.67E-04	↑ cg13047596	↓ *C2orf82*
rs6747645		0.977	2	233571033	A	<u>G</u>	0.637	0.647	0.95	1.39E-04		
rs1022330		0.977	2	233577330	<u>A</u>	G	0.357	0.348	1.05	1.15E-04		
rs6714245		0.977	2	233580505	T	<u>C</u>	0.643	0.652	0.95	1.12E-04		
rs6746294		0.977	2	233583050	C	<u>G</u>	0.645	0.653	0.95	9.06E-05		
rs13003675		0.977	2	233584109	<u>T</u>	C	0.355	0.346	1.06	6.88E-05		
rs13004406		0.977	2	233584557	A	<u>T</u>	0.642	0.651	0.95	1.49E-04		
rs6745879		0.977	2	233590007	A	<u>G</u>	0.645	0.654	0.95	6.66E-05		
rs6717841		0.977	2	233590255	<u>T</u>	C	0.355	0.346	1.06	6.42E-05		
rs11555646		0.977	2	233599904	A	<u>C</u>	0.645	0.654	0.95	5.80E-05		
rs13013142		0.977	2	233600606	A	<u>G</u>	0.645	0.654	0.95	5.73E-05		
rs6738386		0.977	2	233602028	T	<u>C</u>	0.645	0.654	0.95	5.06E-05		
rs7606090		0.954	2	233606740	T	<u>C</u>	0.645	0.654	0.95	4.90E-05		
rs3817311		0.977	2	233612557	<u>T</u>	C	0.355	0.346	1.06	5.13E-05		
rs11679079		0.977	2	233612656	<u>T</u>	C	0.355	0.346	1.06	5.29E-05		
rs13011298		0.977	2	233612996	A	<u>G</u>	0.645	0.654	0.95	5.30E-05		
rs12474040		0.977	2	233615345	T	<u>G</u>	0.645	0.654	0.95	5.28E-05		
rs12990821		0.977	2	233617585	C	<u>G</u>	0.645	0.654	0.95	5.09E-05		
rs6748027		1.0	2	233629552	T	<u>C</u>	0.645	0.654	0.95	4.86E-05		
rs1801251		1.0	2	233633460	<u>A</u>	G	0.355	0.346	1.06	4.42E-05		
rs1446308		1.0	2	233639309	T	<u>C</u>	0.645	0.654	0.95	4.67E-05		
rs737028		1.0	2	233640750	T	<u>C</u>	0.644	0.653	0.95	4.74E-05		
rs283486		1.0	2	233644223	A	<u>G</u>	0.645	0.653	0.95	4.73E-05		
rs283476		1.0	2	233650168	A	<u>G</u>	0.657	0.666	0.95	5.09E-05		
rs283475		1.0	2	233654381	<u>T</u>	G	0.342	0.334	1.06	9.06E-05		
rs283474		1.0	2	233654627	A	<u>G</u>	0.656	0.665	0.95	7.96E-05		
rs283471		1.0	2	233656627	<u>A</u>	G	0.342	0.333	1.06	5.30E-05		
rs283469		1.0	2	233656997	<u>T</u>	G	0.353	0.345	1.06	4.45E-05		
rs283468		1.0	2	233658309	<u>T</u>	C	0.342	0.333	1.06	5.26E-05		
rs2674839		1.0	2	233669040	<u>C</u>	G	0.354	0.346	1.06	5.56E-05		
rs1867778		0.977	2	233679644	T	<u>C</u>	0.646	0.655	0.94	4.11E-05		
rs7558609		1.0	2	233700379	<u>A</u>	G	0.354	0.346	1.06	7.06E-05		
rs1446311		1.0	2	233705071	A	<u>G</u>	0.646	0.654	0.95	6.97E-05		
rs11676216*		1.0	2	233706368	T	<u>C</u>	0.646	0.654	0.95	6.78E-05		
rs10933412		1.0	2	233707226	C	<u>G</u>	0.646	0.654	0.95	6.76E-05		
rs3816334		1.0	2	233708806	<u>A</u>	G	0.354	0.346	1.06	6.66E-05		
rs4973054		1.0	2	233710713	C	<u>G</u>	0.646	0.654	0.95	6.82E-05		
rs991873		1.0	2	233711046	<u>A</u>	G	0.354	0.346	1.06	6.89E-05		
rs6437074		1.0	2	233712296	A	<u>G</u>	0.646	0.654	0.95	7.05E-05		
rs2100053		1.0	2	233719516	T	<u>G</u>	0.645	0.653	0.95	8.18E-05		
rs4973055		0.977	2	233720283	T	<u>G</u>	0.639	0.647	0.95	1.07E-04		
rs1947105		1.0	2	233721455	A	<u>G</u>	0.645	0.654	0.95	7.31E-05		
rs7589201		1.0	2	233724536	<u>A</u>	G	0.355	0.346	1.06	7.49E-05		
rs895430		1.0	2	233725483	A	<u>C</u>	0.646	0.655	0.95	9.84E-05		
rs2675971		1.0	2	233726154	<u>A</u>	G	0.355	0.346	1.06	7.31E-05		
rs6963258	rs4140961	0.937	7	31346832	A	<u>T</u>	0.401	0.405	0.95	7.43E-05	↓ cg11554507	-
rs6964113		0.937	7	31347163	<u>C</u>	G	0.598	0.593	1.05	8.16E-05		
rs4140961*		1	7	31349352	<u>A</u>	G	0.597	0.592	1.06	6.05E-05		
rs7104929*	rs7104929	1.0	11	784340	C	<u>G</u>	0.512	0.526	0.94	**7.89E-06**	↓cg20225915	↑ *PIDD1* ↓ *PNPLA2*
rs4963153		0.941	11	791462	<u>A</u>	G	0.492	0.478	1.06	**1.04E-05**		
rs7479101*	rs7479101	1.0	11	802115	A	<u>G</u>	0.317	0.33	0.93	**5.90E-06**		
rs10902222		0.896	11	810882	<u>T</u>	G	0.697	0.683	1.07	**2.03E-06**		
rs10902221	rs4131364	0.941	11	802379	T	<u>C</u>	0.478	0.492	0.93	**9.70E-07**		
rs6597981		0.941	11	803017	A	<u>G</u>	0.471	0.485	0.94	**2.77E-06**		
rs4131364*		1.0	11	812188	<u>A</u>	G	0.517	0.502	1.07	**1.60E-06**		
rs1054252	rs11600377	0.973	11	68772072	A	<u>G</u>	0.27	0.281	0.94	3.86E-05	↑ cg04464446	↑ *MRPL21, MRGPRD* ↓ *IGHMBP2*
rs11600377*		1.0	11	68785803	<u>A</u>	G	0.731	0.72	1.06	4.38E-05		

ASM: Allele-specific methylation; [1] Data obtained from the PGC+iPSYCH ADHD GWAS meta-analysis (Demontis et al., 2019); [2]Described in Zhang et al., 2010 and Gibbs et al., 2010; [3]eQTL information for brain tissues; SNP: Single Nucleotide Polymorphism; LD: linkage disequilibrium; Chr: Chromosome; Pos: Position (build hg19); A1: Allele 1; A2: Allele 2; All alleles are reported in the forward strand; Freq A1: Frequency of allele 1; OR: Odds Ratio (calculated on A1); Underlined allele: Risk allele for ADHD; ↑: Hypermethylation/Overexpression; ↓: Hypomethylation/Downexpression; "-": No significant data for the SNP; *: Significant tagSNPs overcoming 5% FDR, the other SNPs are ASM SNPs in LD with these significant tagSNPs; In bold: Significant associations for the association between ASM tagSNPs and ADHD p-values overcoming Bonferroni correction for multiple testing and p-value threshold determined using independent number of tests (GEC).

Table S4. Histone marks for the ASM SNPs correlating with differential methylation of cg22930187 and cg06207804.

SNP			Hippocampus Middle	Substantia Nigra	Anterior Caudate	Cingulate Gyrus	Inferior Temporal Lobe	Angular Gyrus	Dorsolateral Prefrontal Cortex	Germinal Matrix	Fetal Brain Female	Fetal Brain Male
**rs2906458**	Chromatin state	15-state model										
		25-state model										
	Enhancer histone marks	H3K4me1	H3K4me1			H3K4me1		H3K4me1	H3K4me1		H3K4me1	
		H3K27ac							H3K27ac			
	Promoter histone marks	H3K4me3										
		H3K9ac										
**rs7412307**	Chromatin state	15-state model										
		25-state model										
	Enhancer histone marks	H3K4me1										
		H3K27ac	H3K27ac	H3K27ac	H3K27ac	H3K27ac		H3K27ac	H3K27ac			
	Promoter histone marks	H3K4me3	H3K4me3					H3K4me3				
		H3K9ac			H3K9ac							
**rs12410334**	Chromatin state	15-state model	EnhG	TxFlnk	EnhG	TxFlnk	EnhG	EnhG	TxFlnk	EnhG	TxFlnk	EnhG
		25-state model	TxReg	TxReg	TxReg	TxReg	TxReg	TxReg	TxReg	TxEnh5	TxEnh5	TxReg
	Enhancer histone marks	H3K4me1	H3K4me1	H3K4me1	H3K4me1	H3K4me1	H3K4me1	H3K4me1	H3K4me1	H3K4me1	H3K4me1	H3K4me1
		H3K27ac	H3K27ac	H3K27ac	H3K27ac	H3K27ac	H3K27ac	H3K27ac	H3K27ac			
	Promoter histone marks	H3K4me3	H3K4me3	H3K4me3	H3K4me3	H3K4me3	H3K4me3	H3K4me3	H3K4me3	H3K4me3	H3K4me3	
		H3K9ac		H3K9ac	H3K9ac	H3K9ac	H3K9ac	H3K9ac	H3K9ac			

ASM: Allele-specific methylation; H3K4me1 and H3K27ac: Histone marks related to enhancer regions; H3K4me3 and H3K9ac: Histone marks related to promoter regions; EnhG: Genic enhancer; TxFlnk: Transcription at gene 5' and 3'; TxReg: Transcribed and regulatory (Promoter/Enhancer); TxEnh5: Transcribed 5' preferential and enhancers; Gray cells: Experiments from Roadmap are not available.

Table S5. Histone marks for the ASM SNPs correlating with differential methylation of cg13047596.

SNP			Hippocampus Middle	Substantia Nigra	Anterior Caudate	Cingulate Gyrus	Inferior Temporal Lobe	Angular Gyrus	Dorsolateral Prefrontal Cortex	Germinal Matrix	Fetal Brain Female	Fetal Brain Male
**rs2012221**	Chromatin state	15-state model										
		25-state model										
	Enhancer histone marks	H3K4me1	H3K4me1	H3K4me1	H3K4me1	H3K4me1		H3K4me1	H3K4me1			
		H3K27ac					H3K27ac					
	Promoter histone marks	H3K4me3						H3K4me3	H3K4me3			
		H3K9ac		H3K9ac	H3K9ac		H3K9ac	H3K9ac				
**rs6747645**	Chromatin state	15-state model										
		25-state model										
	Enhancer histone marks	H3K4me1										
		H3K27ac										
	Promoter histone marks	H3K4me3										
		H3K9ac						H3K9ac				
**rs1022330**	Chromatin state	15-state model										
		25-state model										
	Enhancer histone marks	H3K4me1										
		H3K27ac										
	Promoter histone marks	H3K4me3										
		H3K9ac										
**rs6714245**	Chromatin state	15-state model										
		25-state model										
	Enhancer histone marks	H3K4me1	H3K4me1	H3K4me1	H3K4me1		H3K4me1	H3K4me1	H3K4me1			
		H3K27ac										
	Promoter histone marks	H3K4me3										
		H3K9ac										
**rs6746294**	Chromatin state	15-state model										
		25-state model										
	Enhancer histone marks	H3K4me1	H3K4me1	H3K4me1	H3K4me1			H3K4me1	H3K4me1			
		H3K27ac										
	Promoter histone marks	H3K4me3										
		H3K9ac										
**rs13003675**	Chromatin state	15-state model										
		25-state model										
	Enhancer histone marks	H3K4me1	H3K4me1	H3K4me1	H3K4me1			H3K4me1	H3K4me1			
		H3K27ac										
	Promoter histone marks	H3K4me3										
		H3K9ac										
**rs13004406**	Chromatin state	15-state model										
		25-state model										
	Enhancer histone marks	H3K4me1			H3K4me1			H3K4me1				
		H3K27ac										

	Promoter histone marks	H3K4me3										
		H3K9ac										
**rs6745879**	Chromatin state	15-state model										
		25-state model										
	Enhancer histone marks	H3K4me1										
		H3K27ac										
	Promoter histone marks	H3K4me3										
		H3K9ac										
**rs6717841**	Chromatin state	15-state model										
		25-state model										
	Enhancer histone marks	H3K4me1										
		H3K27ac										
	Promoter histone marks	H3K4me3										
		H3K9ac										
**rs11555646**	Chromatin state	15-state model										
		25-state model										
	Enhancer histone marks	H3K4me1	H3K4me1						H3K4me1			
		H3K27ac										
	Promoter histone marks	H3K4me3										
		H3K9ac										
**rs13013142**	Chromatin state	15-state model										
		25-state model										
	Enhancer histone marks	H3K4me1							H3K4me1			
		H3K27ac										
	Promoter histone marks	H3K4me3										
		H3K9ac										
**rs6738386**	Chromatin state	15-state model										
		25-state model										
	Enhancer histone marks	H3K4me1										
		H3K27ac										
	Promoter histone marks	H3K4me3										
		H3K9ac										
**rs7606090**	Chromatin state	15-state model										
		25-state model										
	Enhancer histone marks	H3K4me1		H3K4me1								
		H3K27ac										
	Promoter histone marks	H3K4me3										
		H3K9ac										
**rs3817311**	Chromatin state	15-state model										
		25-state model										
	Enhancer histone marks	H3K4me1										
		H3K27ac										
	Promoter histone	H3K4me3										

	marks	H3K9ac										
rs11679079	Chromatin state	15-state model										
		25-state model										
	Enhancer histone	H3K4me1										
	marks	H3K27ac										
	Promoter histone	H3K4me3										
	marks	H3K9ac										
rs13011298	Chromatin state	15-state model										
		25-state model										
	Enhancer histone	H3K4me1										
	marks	H3K27ac										
	Promoter histone	H3K4me3										
	marks	H3K9ac										
rs12474040	Chromatin state	15-state model										
		25-state model										
	Enhancer histone	H3K4me1										
	marks	H3K27ac										
	Promoter histone	H3K4me3										
	marks	H3K9ac			H3K9ac							
rs12990821	Chromatin state	15-state model										
		25-state model										
	Enhancer histone	H3K4me1										
	marks	H3K27ac										
	Promoter histone	H3K4me3										
	marks	H3K9ac			H3K9ac							
rs6748027	Chromatin state	15-state model										
		25-state model										
	Enhancer histone	H3K4me1										
	marks	H3K27ac						H3K27ac				
	Promoter histone	H3K4me3										
	marks	H3K9ac		H3K9ac								
rs1801251	Chromatin state	15-state model										
		25-state model										
	Enhancer histone	H3K4me1										
	marks	H3K27ac										
	Promoter histone	H3K4me3										
	marks	H3K9ac			H3K9ac							
rs1446308	Chromatin state	15-state model										
		25-state model										
	Enhancer histone	H3K4me1	H3K4me1	H3K4me1	H3K4me1	H3K4me1	H3K4me1	H3K4me1				
	marks	H3K27ac										
	Promoter histone	H3K4me3										
	marks	H3K9ac										

**rs737028**	Chromatin state	15-state model										
		25-state model										
	Enhancer histone marks	H3K4me1	H3K4me1	H3K4me1	H3K4me1		H3K4me1	H3K4me1	H3K4me1			
		H3K27ac										
	Promoter histone marks	H3K4me3										
		H3K9ac										
**rs283486**	Chromatin state	15-state model		Enh				Enh				
		25-state model		EnhW2	EnhW2	EnhW2	EnhW2	EnhAF	EnhW2			
	Enhancer histone marks	H3K4me1	H3K4me1	H3K4me1	H3K4me1	H3K4me1		H3K4me1	H3K4me1			
		H3K27ac		H3K27ac								
	Promoter histone marks	H3K4me3										
		H3K9ac		H3K9ac	H3K9ac							
**rs283476**	Chromatin state	15-state model										
		25-state model										
	Enhancer histone marks	H3K4me1		H3K4me1		H3K4me1						
		H3K27ac	H3K27ac	H3K27ac	H3K27ac	H3K27ac						
	Promoter histone marks	H3K4me3										
		H3K9ac			H3K9ac							
**rs283475**	Chromatin state	15-state model										
		25-state model										
	Enhancer histone marks	H3K4me1	H3K4me1									
		H3K27ac	H3K27ac	H3K27ac	H3K27ac		H3K27ac	H3K27ac				
	Promoter histone marks	H3K4me3										
		H3K9ac			H3K9ac							
**rs283474**	Chromatin state	15-state model										
		25-state model										
	Enhancer histone marks	H3K4me1	H3K4me1									
		H3K27ac	H3K27ac	H3K27ac	H3K27ac		H3K27ac	H3K27ac				
	Promoter histone marks	H3K4me3										
		H3K9ac			H3K9ac							
**rs283471**	Chromatin state	15-state model										
		25-state model										
	Enhancer histone marks	H3K4me1										
		H3K27ac					H3K27ac					
	Promoter histone marks	H3K4me3										
		H3K9ac										
**rs283469**	Chromatin state	15-state model										
		25-state model										
	Enhancer histone marks	H3K4me1			H3K4me1							
		H3K27ac	H3K27ac				H3K27ac	H3K27ac				
	Promoter histone marks	H3K4me3										
		H3K9ac										
**rs283468**	Chromatin state	15-state model										

	Chromatin state	25-state model										
	Enhancer histone marks	H3K4me1			H3K4me1							
		H3K27ac	H3K27ac	H3K27ac				H3K27ac				
	Promoter histone marks	H3K4me3										
		H3K9ac										
**rs2674839**	Chromatin state	15-state model										
		25-state model										
	Enhancer histone marks	H3K4me1										
		H3K27ac										
	Promoter histone marks	H3K4me3										
		H3K9ac										
**rs1867778**	Chromatin state	15-state model				EnhG						
		25-state model										
	Enhancer histone marks	H3K4me1	H3K4me1	H3K4me1		H3K4me1	H3K4me1		H3K4me1			
		H3K27ac						H3K27ac				
	Promoter histone marks	H3K4me3										
		H3K9ac					H3K9ac					
**rs7558609**	Chromatin state	15-state model	EnhG	Enh				EnhG				
		25-state model	TxEnh3	TxEnh3	TxEnh3	TxEnh3	TxEnh3	TxEnh3	TxEnh3			
	Enhancer histone marks	H3K4me1	H3K4me1	H3K4me1	H3K4me1	H3K4me1	H3K4me1	H3K4me1	H3K4me1			
		H3K27ac	H3K27ac	H3K27ac	H3K27ac	H3K27ac	H3K27ac	H3K27ac	H3K27ac			
	Promoter histone marks	H3K4me3										
		H3K9ac		H3K9ac	H3K9ac	H3K9ac		H3K9ac				
**rs1446311**	Chromatin state	15-state model		EnhG								
		25-state model										
	Enhancer histone marks	H3K4me1	H3K4me1	H3K4me1	H3K4me1		H3K4me1	H3K4me1	H3K4me1			
		H3K27ac	H3K27ac	H3K27ac	H3K27ac	H3K27ac	H3K27ac	H3K27ac	H3K27ac			
	Promoter histone marks	H3K4me3										
		H3K9ac		H3K9ac	H3K9ac		H3K9ac	H3K9ac				
**rs11676216**	Chromatin state	15-state model										
		25-state model										
	Enhancer histone marks	H3K4me1	H3K4me1									
		H3K27ac		H3K27ac	H3K27ac		H3K27ac	H3K27ac	H3K27ac			
	Promoter histone marks	H3K4me3										
		H3K9ac			H3K9ac							
**rs10933412**	Chromatin state	15-state model										
		25-state model										
	Enhancer histone marks	H3K4me1			H3K4me1							
		H3K27ac	H3K27ac	H3K27ac	H3K27ac	H3K27ac	H3K27ac	H3K27ac	H3K27ac			
	Promoter histone marks	H3K4me3										
		H3K9ac			H3K9ac							
**rs3816334**	Chromatin state	15-state model					EnhG					
		25-state model			TxEnh3		TxEnh3	TxEnh3	TxEnh3			

	Enhancer histone marks	H3K4me1										
		H3K27ac	H3K27ac		H3K27ac	H3K27ac	H3K27ac	H3K27ac	H3K27ac			
	Promoter histone marks	H3K4me3										
		H3K9ac			H3K9ac		H3K9ac	H3K9ac				
**rs4973054**	Chromatin state	15-state model										
		25-state model										
	Enhancer histone marks	H3K4me1										
		H3K27ac			H3K27ac	H3K27ac		H3K27ac				
	Promoter histone marks	H3K4me3										
		H3K9ac			H3K9ac			H3K9ac				
**rs991873**	Chromatin state	15-state model										
		25-state model										
	Enhancer histone marks	H3K4me1			H3K4me1							
		H3K27ac			H3K27ac	H3K27ac		H3K27ac				
	Promoter histone marks	H3K4me3										
		H3K9ac			H3K9ac			H3K9ac				
**rs6437074**	Chromatin state	15-state model										
		25-state model										
	Enhancer histone marks	H3K4me1		H3K4me1	H3K4me1	H3K4me1			H3K4me1			
		H3K27ac	H3K27ac	H3K27ac	H3K27ac	H3K27ac		H3K27ac				
	Promoter histone marks	H3K4me3										
		H3K9ac		H3K9ac	H3K9ac			H3K9ac				
**rs2100053**	Chromatin state	15-state model										
		25-state model										
	Enhancer histone marks	H3K4me1	H3K4me1	H3K4me1	H3K4me1			H3K4me1				
		H3K27ac	H3K27ac	H3K27ac	H3K27ac	H3K27ac	H3K27ac	H3K27ac	H3K27ac			
	Promoter histone marks	H3K4me3										
		H3K9ac		H3K9ac	H3K9ac			H3K9ac				
**rs4973055**	Chromatin state	15-state model										
		25-state model										
	Enhancer histone marks	H3K4me1	H3K4me1		H3K4me1							
		H3K27ac	H3K27ac	H3K27ac	H3K27ac	H3K27ac	H3K27ac	H3K27ac	H3K27ac			
	Promoter histone marks	H3K4me3										
		H3K9ac		H3K9ac				H3K9ac				
**rs1947105**	Chromatin state	15-state model										
		25-state model										
	Enhancer histone marks	H3K4me1										
		H3K27ac	H3K27ac	H3K27ac	H3K27ac	H3K27ac		H3K27ac	H3K27ac			
	Promoter histone marks	H3K4me3										
		H3K9ac		H3K9ac	H3K9ac							
**rs7589201**	Chromatin state	15-state model										
		25-state model										
	Enhancer histone	H3K4me1										

	marks	H3K27ac	H3K27ac	H3K27ac		H3K27ac						
	Promoter histone marks	H3K4me3										
		H3K9ac			H3K9ac							
**rs895430**	Chromatin state	15-state model										
		25-state model										
	Enhancer histone marks	H3K4me1										
		H3K27ac	H3K27ac	H3K27ac		H3K27ac						
	Promoter histone marks	H3K4me3										
		H3K9ac			H3K9ac							
**rs2675971**	Chromatin state	15-state model										
		25-state model										
	Enhancer histone marks	H3K4me1										
		H3K27ac		H3K27ac								
	Promoter histone marks	H3K4me3										
		H3K9ac										

ASM: Allele-specific methylation; H3K4me1 and H3K27ac: Histone marks related to enhancer regions; H3K4me3 and H3K9ac: Histone marks related to promoter regions; Enh: Enhancers; EnhW2: Weak Enhancer 2; EnhG: Genic enhancer; EnhAF: Active enhancer Flank; TxEnh3: Transcribed 3' preferential and enhancers; Gray cells: Experiments from Roadmap are not available.

Table S6. Histone marks for the ASM SNPs correlating with differential methylation of cg11554507.

SNP			Hippocampus Middle	Substantia Nigra	Anterior Caudate	Cingulate Gyrus	Inferior Temporal Lobe	Angular Gyrus	Dorsolateral Prefrontal Cortex	Germinal Matrix	Fetal Brain Female	Fetal Brain Male
**rs6963258**	Chromatin state	15-state model										
		25-state model										
	Enhancer histone marks	H3K4me1									H3K4me1	H3K4me1
		H3K27ac										
	Promoter histone marks	H3K4me3										
		H3K9ac										
**rs6964113**	Chromatin state	15-state model										
		25-state model										
	Enhancer histone marks	H3K4me1									H3K4me1	H3K4me1
		H3K27ac										
	Promoter histone marks	H3K4me3										
		H3K9ac										
**rs4140961**	Chromatin state	15-state model								Enh	Enh	Enh
		25-state model										EnhAc
	Enhancer histone marks	H3K4me1								H3K4me1	H3K4me1	H3K4me1
		H3K27ac										
	Promoter histone marks	H3K4me3										
		H3K9ac										

ASM: Allele-specific methylation; H3K4me1 and H3K27ac: Histone marks related to enhancer regions; H3K4me3 and H3K9ac: Histone marks related to promoter regions; Enh: Enhancers; EnhAc: Primary H3K27ac possible enhancer; Gray cells: Experiments from Roadmap are not available.

Table S7. Histone marks for the ASM SNPs correlating with differential methylation of cg20225915.

SNP			Hippocampus Middle	Substantia Nigra	Anterior Caudate	Cingulate Gyrus	Inferior Temporal Lobe	Angular Gyrus	Dorsolateral Prefrontal Cortex	Germinal Matrix	Fetal Brain Female	Fetal Brain Male
**rs7104929**	Chromatin state	15-state model									Enh	Enh
		25-state model									EnhW2	EnhAc
	Enhancer histone marks	H3K4me1	H3K4me1		H3K4me1	H3K4me1	H3K4me1	H3K4me1	H3K4me1		H3K4me1	H3K4me1
		H3K27ac	H3K27ac	H3K27ac	H3K27ac	H3K27ac	H3K27ac	H3K27ac	H3K27ac			
	Promoter histone marks	H3K4me3							H3K4me3			
		H3K9ac			H3K9ac		H3K9ac		H3K9ac			
**rs4963153**	Chromatin state	15-state model										
		25-state model										
	Enhancer histone marks	H3K4me1							H3K4me1			H3K4me1
		H3K27ac	H3K27ac	H3K27ac	H3K27ac	H3K27ac	H3K27ac		H3K27ac			
	Promoter histone marks	H3K4me3										
		H3K9ac			H3K9ac			H3K9ac	H3K9ac			
**rs7479101**	Chromatin state	15-state model										
		25-state model										
	Enhancer histone marks	H3K4me1							H3K4me1			
		H3K27ac						H3K27ac	H3K27ac			
	Promoter histone marks	H3K4me3										
		H3K9ac			H3K9ac	H3K9ac	H3K9ac	H3K9ac				
**rs10902221**	Chromatin state	15-state model										
		25-state model										
	Enhancer histone marks	H3K4me1							H3K4me1			H3K4me1
		H3K27ac	H3K27ac					H3K27ac	H3K27ac			
	Promoter histone marks	H3K4me3										
		H3K9ac			H3K9ac	H3K9ac	H3K9ac	H3K9ac				
**rs6597981**	Chromatin state	15-state model										
		25-state model										
	Enhancer histone marks	H3K4me1						H3K4me1	H3K4me1	H3K4me1		H3K4me1
		H3K27ac	H3K27ac			H3K27ac	H3K27ac	H3K27ac	H3K27ac			
	Promoter histone marks	H3K4me3					H3K4me3					
		H3K9ac		H3K9ac	H3K9ac	H3K9ac	H3K9ac	H3K9ac	H3K9ac			
**rs10902222**	Chromatin state	15-state model										
		25-state model										
	Enhancer histone marks	H3K4me1	H3K4me1	H3K4me1	H3K4me1	H3K4me1	H3K4me1	H3K4me1	H3K4me1	H3K4me1	H3K4me1	H3K4me1
		H3K27ac	H3K27ac	H3K27ac	H3K27ac	H3K27ac	H3K27ac	H3K27ac	H3K27ac			
	Promoter histone marks	H3K4me3	H3K4me3	H3K4me3	H3K4me3	H3K4me3	H3K4me3	H3K4me3	H3K4me3	H3K4me3	H3K4me3	H3K4me3
		H3K9ac		H3K9ac	H3K9ac	H3K9ac	H3K9ac	H3K9ac	H3K9ac			
**rs4131364**	Chromatin state	15-state model	EnhG			EnhG			EnhG	EnhG		
		25-state model	TxEnh5		TxEnh5	TxEnh5	TxEnh5	TxEnh5	TxEnh5	TxEnh5	TxEnh5	
	Enhancer histone marks	H3K4me1	H3K4me1	H3K4me1	H3K4me1	H3K4me1	H3K4me1	H3K4me1	H3K4me1	H3K4me1	H3K4me1	H3K4me1
		H3K27ac	H3K27ac	H3K27ac	H3K27ac	H3K27ac	H3K27ac	H3K27ac	H3K27ac			
	Promoter histone	H3K4me3	H3K4me3				H3K4me3		H3K4me3	H3K4me3	H3K4me3	

marks	H3K9ac		H3K9ac	H3K9ac		H3K9ac	H3K9ac	H3K9ac			

ASM: Allele-specific methylation; H3K4me1 and H3K27ac: Histone marks related to enhancer regions; H3K4me3 and H3K9ac: Histone marks related to promoter regions; Enh: Enhancers; EnhW2: Weak Enhancer 2; EnhAc: Primary H3K27ac possible enhancer; EnhG: Genic enhancer; TxEnh5: Transcribed 5' preferential and enhancers; Gray cells: Experiments from Roadmap are not available.

Table S8. Histone marks for the ASM SNPs correlating with differential methylation of cg04464446.

SNP			Hippocampus Middle	Substantia Nigra	Anterior Caudate	Cingulate Gyrus	Inferior Temporal Lobe	Angular Gyrus	Dorsolateral Prefrontal Cortex	Germinal Matrix	Fetal Brain Female	Fetal Brain Male
**rs1054252**	Chromatin state	15-state model										
		25-state model										
	Enhancer histone marks	H3K4me1							H3K4me1			
		H3K27ac	H3K27ac			H3K27ac			H3K27ac			
	Promoter histone marks	H3K4me3										
		H3K9ac										
**rs11600377**	Chromatin state	15-state model										
		25-state model										
	Enhancer histone marks	H3K4me1										
		H3K27ac										
	Promoter histone marks	H3K4me3										
		H3K9ac										

ASM:Allele-specific methylation; H3K4me1 and H3K27ac: Histone marks related to enhancer regions; H3K4me3 and H3K9ac: Histone marks related to promoter regions; Gray cells: Experiments from Roadmap are not available.

Table S9. MetaXcan prediction of gene expression effects on ADHD for multiple brain tissues.

Gene	Brain tissue*	Z-score	p-value	N SNPs in model	N SNPs used	N ASM SNPs	Predicted R^2
ARTN	Caudate basal ganglia	1.54	1.20E-01	19	16	0	0.10
	Cerebellar hemisphere	4.19	**2.50E-05**	15	15	4	0.37
	Cerebellum	3.57	**3.50E-04**	31	31	5	0.36
	Cortex	3.94	**8.10E-05**	5	4	2	0.14
	Frontal cortex	1.42	1.50E-01	29	24	2	0.09
	Hippocampus	1.55	1.10E-01	140	129	2	0.16
C2orf82	Dorsolateral prefrontal cortex	-3.50	**4.50E-04**	58	41	9	0.43
	Amygdala	-3.07	**2.00E-03**	92	89	42	0.40
	Anterior cingulate cortex	-3.55	**3.00E-04**	11	11	7	0.19
	Caudate basal ganglia	-3.54	**3.00E-04**	11	11	8	0.44
	Cerebellar hemisphere	-3.64	**2.00E-04**	37	35	6	0.19
	Cerebellum	-3.50	**4.00E-04**	30	30	7	0.28
	Cortex	-3.46	**5.00E-04**	59	54	7	0.27
	Frontal cortex	-3.59	**3.00E-04**	29	28	6	0.45
	Hippocampus	-3.40	**6.00E-04**	25	23	8	0.24
	Hypothalamus	-3.37	**7.00E-04**	30	28	13	0.17
	Nucleus accumbens basal ganglia	-3.59	**3.00E-04**	29	29	11	0.33
	Putamen basal ganglia	-3.20	**1.00E-03**	52	43	9	0.52
PIDD1	Dorsolateral prefrontal cortex	4.71	**2.41E-06**	4	2	1	0.06
	Cerebellar hemisphere	5.00	**4.20E-07**	32	27	4	0.53
	Cerebellum	5.37	**7.60E-08**	36	27	5	0.49
	Cortex	3.57	**3.40E-04**	64	47	2	0.03

ASM: Allele-specific methylation; *ADHD prediction models were only available for some tissues and genes; Z-score: Number of standard deviations change in gene expression in ADHD; p-value: Significance of the association between predicted expression levels and ADHD; N SNPs in model: Number of SNPs used in the training of prediction models for each gene; N SNPs used: Number of SNPs used from the ADHD GWAS meta-analysis summary statistics; N ASM SNPs: Number of ASM SNPs included in the model; Predicted R^2: Correlation between the predicted and observed gene expression during prediction model training; In bold: Significant p-values overcoming Bonferroni correction for multiple testing.

Table S10. Correlations with sub-cortical brain volumes of the ASM SNPs associated with ADHD.

SNP	ASM for CpG site	A1	A2	Nucleus Accumbens		Amygdala		Caudate nucleus		Hippocampus		Pallidum		Putamen		Thalamus	
				Effect	p-value	Effect	p-value	Effect	p-value	Effect	p-value	Effect	p-value	Effect	p-value	Effect	p-value
rs2906458*	cg22930187 cg06207804	A	G	0.2	0.885	0.7	0.784	0.8	0.881	-4.1	0.442	3.9	0.073	8.9	0.191	3.5	0.628
rs7412307*		C	G	?	?	?	?	?	?	?	?	?	?	?	?	?	?
rs12410334		A	C	-0.6	0.651	2.9	0.341	-1.6	0.795	-3.0	0.624	3.9	0.119	3.9	0.619	-4.0	0.623
rs2012221	cg13047596	T	C	2.5	**0.041**	1.7	0.494	10.8	**0.040**	-2.5	0.618	0.2	0.923	8.2	0.202	-4.9	0.471
rs6747645		A	G	2.5	**0.030**	1.8	0.473	11.1	**0.035**	-2.7	0.589	0.5	0.812	8.5	0.187	-4.6	0.496
rs1022330		A	G	2.5	**0.029**	1.8	0.479	10.8	**0.038**	-2.6	0.609	0.2	0.908	8.2	0.200	-4.8	0.471
rs6714245		T	C	2.5	**0.038**	1.8	0.470	10.7	**0.040**	-2.8	0.577	0.3	0.880	8.1	0.202	-4.9	0.467
rs6746294		C	G	?	?	?	?	?	?	?	?	?	?	?	?	?	?
rs13003675		T	C	2.5	**0.036**	1.8	0.465	10.5	**0.044**	-2.2	0.663	0.3	0.897	7.9	0.220	-4.9	0.467
rs13004406		A	T	?	?	?	?	?	?	?	?	?	?	?	?	?	?
rs6745879		A	G	2.5	**0.031**	1.8	0.468	10.6	**0.040**	-2.7	0.585	0.3	0.881	8.1	0.201	-4.9	0.456
rs6717841		T	C	2.4	**0.044**	2.2	0.377	10.1	0.052	-2.4	0.628	0.2	0.941	7.1	0.268	-4.8	0.471
rs11555646		A	C	2.5	**0.028**	1.7	0.478	10.6	**0.039**	-2.7	0.586	0.2	0.923	7.9	0.207	-5.1	0.435
rs13013142		A	G	2.5	**0.028**	1.7	0.478	10.6	**0.039**	-2.7	0.587	0.2	0.922	7.9	0.207	-5.1	0.435
rs6738386		T	C	2.5	**0.028**	1.8	0.476	10.6	**0.039**	-2.7	0.587	0.2	0.920	7.9	0.208	-5.1	0.435
rs7606090		T	C	2.5	**0.027**	1.8	0.470	10.7	**0.037**	-2.7	0.580	0.2	0.912	8.0	0.202	-5.2	0.428
rs3817311		T	C	2.5	**0.029**	1.8	0.472	10.6	**0.038**	-2.7	0.577	0.2	0.915	7.8	0.212	-5.5	0.405
rs11679079		T	C	2.5	**0.029**	1.8	0.472	10.6	**0.038**	-2.7	0.576	0.2	0.916	7.8	0.213	-5.5	0.405
rs13011298		A	G	2.5	**0.029**	1.8	0.472	10.6	**0.037**	-2.7	0.576	0.2	0.916	7.8	0.213	-5.5	0.404
rs12474040		T	G	2.5	**0.029**	1.8	0.473	10.6	**0.038**	-2.8	0.567	0.2	0.918	7.7	0.217	-5.6	0.395
rs12990821		C	G	?	?	?	?	?	?	?	?	?	?	?	?	?	?
rs6748027		T	C	2.5	**0.025**	1.6	0.512	10.5	**0.040**	-3.0	0.536	0.2	0.938	7.6	0.224	-5.5	0.396
rs1801251		A	G	2.5	**0.025**	1.6	0.516	10.5	**0.040**	-3.1	0.531	0.2	0.940	7.6	0.224	-5.6	0.394
rs1446308		T	C	2.5	**0.025**	1.6	0.518	10.5	**0.040**	-3.1	0.532	0.2	0.937	7.6	0.222	-5.5	0.398
rs737028		T	C	2.5	**0.024**	1.6	0.519	10.5	**0.040**	-3.1	0.530	0.2	0.933	7.7	0.218	-5.4	0.404
rs283486		A	G	2.6	**0.023**	1.6	0.517	10.5	**0.039**	-3.1	0.525	0.1	0.944	8.0	0.201	-5.2	0.427
rs283476		A	G	2.1	0.068	2.6	0.301	10.5	**0.041**	-2.1	0.671	-0.7	0.725	6.5	0.307	-3.2	0.634
rs283475		T	G	2.2	0.065	2.6	0.289	10.4	**0.044**	-2.1	0.665	-0.7	0.716	6.4	0.311	-3.2	0.626
rs283474		A	G	2.2	0.065	2.6	0.301	10.5	**0.042**	-2.1	0.664	-0.7	0.714	6.5	0.304	-3.2	0.633
rs283471		A	G	2.2	0.062	2.9	0.252	10.7	**0.039**	-2.1	0.676	-0.7	0.736	6.3	0.318	-2.9	0.667
rs283469		T	G	2.6	**0.025**	1.6	0.506	10.3	**0.044**	-3.0	0.541	0.1	0.974	8.4	0.179	-5.0	0.449
rs283468		T	C	2.2	0.064	2.6	0.299	10.5	**0.042**	-2.2	0.659	-0.8	0.707	6.5	0.308	-3.2	0.630
rs2674839		C	G	?	?	?	?	?	?	?	?	?	?	?	?	?	?
rs1867778		T	C	2.5	**0.030**	1.6	0.516	10.3	**0.044**	-3.2	0.508	0.1	0.973	7.9	0.203	-5.8	0.373
rs7558609		A	G	2.6	**0.019**	1.5	0.537	10.6	**0.037**	-3.4	0.484	0.1	0.974	8.0	0.202	-6.4	0.326
rs1446311		A	G	2.6	**0.020**	1.5	0.526	10.3	**0.043**	-3.1	0.522	0.1	0.967	7.9	0.202	-6.1	0.348
rs11676216*		T	C	2.6	**0.020**	1.5	0.527	10.5	**0.039**	-2.9	0.555	0.0	0.992	8.1	0.194	-6.1	0.348
rs10933412		C	G	?	?	?	?	?	?	?	?	?	?	?	?	?	?
rs3816334		A	G	2.6	**0.019**	1.6	0.508	10.4	**0.042**	-3.0	0.536	0.1	0.959	8.1	0.194	-6.2	0.339
rs4973054		C	G	?	?	?	?	?	?	?	?	?	?	?	?	?	?
rs991873		A	G	2.6	**0.019**	1.6	0.507	10.4	**0.042**	-3.0	0.533	0.1	0.960	8.1	0.194	-6.3	0.337
rs6437074		A	G	2.6	**0.020**	1.7	0.483	9.9	0.052	-3.0	0.536	0.1	0.948	7.8	0.210	-6.6	0.316
rs2100053		T	G	2.6	**0.020**	1.6	0.524	10.1	**0.047**	-3.2	0.514	0.1	0.952	7.9	0.204	-6.0	0.358
rs4973055		T	G	2.6	**0.024**	2.1	0.398	9.6	0.063	-3.1	0.526	0.3	0.881	7.8	0.214	-5.0	0.444
rs1947105		A	G	2.6	**0.021**	1.6	0.499	10.1	**0.048**	-3.2	0.508	0.1	0.959	7.7	0.213	-6.0	0.361
rs7589201		A	G	2.6	**0.020**	1.6	0.519	10.2	**0.046**	-3.2	0.507	0.1	0.956	7.8	0.209	-5.9	0.363
rs895430		A	C	2.6	**0.020**	1.6	0.517	10.2	**0.045**	-3.2	0.510	0.1	0.951	7.8	0.208	-5.9	0.367
rs2675971		A	G	2.6	**0.019**	1.6	0.516	10.2	**0.045**	-3.1	0.522	0.2	0.937	7.9	0.206	-5.8	0.375
rs6963258	cg11554507	A	T	?	?	?	?	?	?	?	?	?	?	?	?	?	?
rs6964113		C	G	?	?	?	?	?	?	?	?	?	?	?	?	?	?
rs4140961*		A	G	1.6	0.147	1.7	0.467	1.9	0.700	7.0	0.147	2.2	0.273	5.8	0.341	13.5	**0.035**
rs7104929*	cg20225915	C	G	?	?	?	?	?	?	?	?	?	?	?	?	?	?
rs4963153		A	G	0.1	0.931	1.7	0.514	-2.4	0.643	-0.6	0.899	0.5	0.795	-3.5	0.588	8.0	0.232
rs7479101*		A	G	0.4	0.766	2.5	0.365	-2.0	0.733	-1.4	0.808	0.6	0.798	-1.7	0.814	-2.1	0.777
rs10902222		T	G	0.5	0.715	1.6	0.557	-2.0	0.727	-2.4	0.668	0.5	0.840	-2.1	0.761	-2.6	0.726
rs10902221		T	C	0.1	0.940	1.4	0.574	-1.2	0.825	-2.1	0.681	0.6	0.773	2.8	0.663	2.9	0.662
rs6597981		A	G	0.2	0.881	1.6	0.530	-0.7	0.890	-1.9	0.714	0.7	0.734	3.4	0.596	3.6	0.593
rs4131364*		A	G	0.1	0.941	1.2	0.622	-0.9	0.862	-3.3	0.511	0.6	0.749	0.0	0.998	0.7	0.915
rs1054252	cg04464446	A	G	-2.5	**0.037**	-1.2	0.651	-11.2	**0.038**	-2.8	0.587	-1.5	0.477	-3.6	0.583	-1.7	0.804
rs11600377*		A	G	-2.5	**0.038**	-0.9	0.741	-11.7	**0.030**	-2.9	0.579	-1.9	0.375	-3.7	0.573	-2.2	0.751

ASM: Allele-specific methylation; A1: Allele 1; A2: Allele 2; All alleles are reported in the forward strand; Effect: Effect sizes are given in units of mm3 per risk allele; *: Significant tagSNPs overcoming 5% FDR, the other SNPs are ASM SNPs in LD with these significant tagSNPs; Underlined allele: Risk allele for ADHD; In bold: Nominally significant p-values; "?": No values retrieved for these SNPs.

Table S11. Copy number variants overlapping with the highlighted genes for ADHD.

Gene	Variant	Type	Sex	Inheritance	Phenotype(s)
ARTN	1:36831723-61417768	Deletion	46XX	Unknown	Hypoplasia of the corpus callosum, Intellectual disability
	1:41126365-45133712	Deletion	46XY	De novo constitutive	Congenital microcephaly
	1:43827703-45107379	Deletion	46XX	De novo constitutive	Autism, Intellectual disability
	1:43870211-45107379	Deletion	46XX	De novo constitutive	Autism, Delayed speech and language development, Intellectual disability
C2orf82	2:216527137-242783384	Duplication	46XX	De novo constitutive	Intellectual disability
	2:217860965-242783384	Deletion	46XX	Unknown	Hypodysplasia of the corpus callosum
	2:228207994-236247812	Deletion	46XY	Unknown	Intellectual disability
	2:231683468-235003677	Duplication	unknown	De novo constitutive	Aggressive behavior, ADHD, Dysgraphia, Self-injurious behavior
	2:231869040-243014630	Duplication	other	De novo constitutive	Aggressive behavior, Dysarthria, Intellectual disability, Microcephaly, Small for gestational age
	2:232756751-238245191	Duplication	46XY	De novo constitutive	Intellectual disability
	2:232763439-242717043	Duplication	46XY	De novo constitutive	Intellectual disability
	2:232831650-239972530	Deletion	46XY	De novo constitutive	Macrocephaly, Mild global developmental delay
	2:232981279-243007359	Deletion	46XX	De novo constitutive	Aggressive behavior, Behavioral abnormality, Failure to thrive, Global developmental delay, Growth delay, Intrauterine growth
	2:233508667-241070353	Duplication	46XX	De novo constitutive	Intellectual disability, Seizures.
	2:233527432-242852625	Duplication	46XY	De novo mosaic	Delayed speech and language development, Global developmental delay, Motor delay
	2:233613670-233742556	Duplication	46XY	Unknown	Cognitive impairment
PIDD1	11:1-18716078	Duplication	46XX	De novo constitutive	Intellectual disability
	11:1-4061418	Duplication	46XX	Maternally inherited, constitutive in mother	Macrocephaly, Postnatal growth retardation
	11:192372-3653923	Triplication	46XX	De novo constitutive	Intellectual disability
	11:196966-3254236	Duplication	46XY	De novo constitutive	Intellectual disability, Small for gestational age
	11:198510-2261562	Duplication	46XX	De novo constitutive	Global developmental delay
	11:203788-3131418	Duplication	46XX	Unknown	Delayed speech and language development, Global developmental delay
	11:210100-2425271	Duplication	46XY	Imbalance arising from a balanced parental rearrangement	Delayed speech and language development, Muscular hypotonia
	11:210300-3363804	Duplication	46XX	De novo constitutive	Autism
	11:353347-872723	Duplication	46XY	Maternally inherited, constitutive in mother	Delayed speech and language development
	11:662317-1157708	Duplication	46XX	Maternally inherited, constitutive in mother	Global developmental delay

Variant: CNV position (build hg19); Source: DECIPHER (v9.27) database; The reported phenotypes have been restricted to those comorbid to ADHD or neurodevelopmental processes.
